# Bayesian Diallel Analysis Reveals *Mx1*-Dependent and *Mx1*-Independent Effects on Response to Influenza A Virus in Mice

**DOI:** 10.1534/g3.117.300438

**Published:** 2017-11-29

**Authors:** Paul L. Maurizio, Martin T. Ferris, Gregory R. Keele, Darla R. Miller, Ginger D. Shaw, Alan C. Whitmore, Ande West, Clayton R. Morrison, Kelsey E. Noll, Kenneth S. Plante, Adam S. Cockrell, David W. Threadgill, Fernando Pardo-Manuel de Villena, Ralph S. Baric, Mark T. Heise, William Valdar

**Affiliations:** * Curriculum in Bioinformatics and Computational Biology, University of North Carolina at Chapel Hill, North Carolina 27599; † Department of Genetics, University of North Carolina at Chapel Hill, North Carolina 27599; ‡ Department of Epidemiology, University of North Carolina at Chapel Hill, North Carolina 27599; § Department of Microbiology and Immunology, University of North Carolina at Chapel Hill, North Carolina 27599; ** Institute of Genome Sciences and Society, Texas A&M University, College Station, Texas 77433-2470; †† Lineberger Comprehensive Cancer Center, University of North Carolina at Chapel Hill, North Carolina 27599

**Keywords:** treatment response, causal effect, Bayesian mixed model, multiple imputation, multiparental populations, MPP

## Abstract

Influenza A virus (IAV) is a respiratory pathogen that causes substantial morbidity and mortality during both seasonal and pandemic outbreaks. Infection outcomes in unexposed populations are affected by host genetics, but the host genetic architecture is not well understood. Here, we obtain a broad view of how heritable factors affect a mouse model of response to IAV infection using an 8 × 8 diallel of the eight inbred founder strains of the Collaborative Cross (CC). Expanding on a prior statistical framework for modeling treatment response in diallels, we explore how a range of heritable effects modify acute host response to IAV through 4 d postinfection. Heritable effects in aggregate explained ∼57% of the variance in IAV-induced weight loss. Much of this was attributable to a pattern of additive effects that became more prominent through day 4 postinfection and was consistent with previous reports of antiinfluenza myxovirus resistance 1 (*Mx1*) polymorphisms segregating between these strains; these additive effects largely recapitulated haplotype effects observed at the *Mx1* locus in a previous study of the incipient CC, and are also replicated here in a CC recombinant intercross population. Genetic dominance of protective *Mx1* haplotypes was observed to differ by subspecies of origin: relative to the *domesticus* null *Mx1* allele, *musculus* acts dominantly whereas *castaneus* acts additively. After controlling for *Mx1*, heritable effects, though less distinct, accounted for ∼34% of the phenotypic variance. Implications for future mapping studies are discussed.

Pathogenic response to viral infection varies dramatically between individuals infected with the same viral strain and dose, and much of this variation is heritable. The impact of host genetics is evident both on the primary exposure to a virus during early life (Strunk *et al.* 2013) and upon infection with newly emerging viral strains; the latter, where prior immune exposure to a variant viral strain is not cross-protective, being especially common for quickly evolving RNA viruses such as the influenza A virus (IAV) (Morens *et al.* 2010). Pathogenesis induced by IAV, whether contracted during early childhood or later in life, is thus likely to have a significant heritable component. A greater understanding of this heritability should improve our ability to not only identify populations at risk of enhanced morbidity and mortality during an emerging pandemic, but also to identify successful options for treatment.

The past several years have seen significant progress in identifying and characterizing host genes that modulate susceptibility to IAV infection via knockout mouse studies, *in vitro* screens, and studies of primary immunodeficiencies and allelic variants in humans (To *et al.* 2015). In humans, screening for inborn errors identified a major role for interferon regulatory factor 7 (*Irf7*) in modulating the severity of primary IAV infection (Ciancanelli *et al.* 2015), and allelic variation in *Ifitm3*, which was identified in a high-throughput siRNA screen, was associated with differential severity of IAV-infection outcomes during the 2009 H1N1 pandemic (Everitt *et al.* 2012).

Most of our insights into genes modulating host IAV resistance, however, have come from studies on mice. These include studies using knockout mice—which have identified host genetic factors critical to antiviral responses, including *Tlr3* (Hidaka *et al.* 2006) and *Isg15* (Lenschow *et al.* 2007)—and studies that examine differences between laboratory inbred strains. Inbred strain studies were the first to identify the *Myxovirus resistance* (*Mx*) family of proteins as important for host antiviral response (Staeheli *et al.* 1988), and inbred studies have continued to demonstrate the relevance of genetic background to multiple aspects of IAV pathogenesis (Srivastava *et al.* 2009; Alberts *et al.* 2010; Leist *et al.* 2016; Samet and Tompkins 2017).

Yet, despite the identification of clear phenotypic differences between inbred strains, there have been relatively few attempts to dissect the genetic basis of those differences using traditional quantitative trait locus (QTL) mapping approaches such as the use of F2s or backcrosses (although see Boivin *et al.* 2012). This may be, in part, because traditional QTL mapping approaches tend to rely on outbred animals—and when it comes to studying viral pathogenesis, outbreds are in many respects problematic. One important limitation is phenotyping: studying the response to an infection is equivalent to studying the causal effect of an applied treatment in that its strict definition relies on a comparison between otherwise identical individuals subject to infection *vs.* control. But such like-for-like comparisons are biologically and technically challenging to make in an outbred population, where every individual is genetically distinct, and this has undesirable consequences for downstream interpretation: namely, that when genetic determinants of severe IAV pathogenesis are confounded with those influencing baseline phenotypes, the roles of any detected QTL are ambiguous. A related disadvantage of outbreds from the perspective of genetics is the inability to obtain biological replicates, which makes it harder to distinguish which aspects of pathology are stable consequences of genetics *vs.* products of stochastic variability. This is particularly important, since it also makes it almost impossible to follow-up on genuinely extreme responders for additional mechanistic and genetic analysis. Translating strain differences in IAV pathogenesis to meaningful QTL studies ideally requires an experimental paradigm that combines population-level genetic diversity with individual-level replicability.

An exciting opportunity is therefore presented by replicable genetic reference populations, in particular, those based on panels of recombinant inbred (RI) strains. Across a panel of RIs, genetic background varies, providing a basis for QTL mapping; within a RI strain, individuals are genetically identical, providing a basis for replication. The combination allows infection response to be rigorously defined and genomic regions affecting that response to be mapped. It also permits the creation of sophisticated experiments that target a wider range of heritable mechanisms: crossing RIs with each other to form RI intercrosses (RIXs), or crossing them with outside strains, produces replicable systems capable of distinguishing, for example, additive, dominance, and parent-of-origin effects, among others (*e.g.*, Xiao *et al.* 1995; Hua *et al.* 2002, 2003; Kollipara *et al.* 2002; Threadgill *et al.* 2002; Mei *et al.* 2005; Gonzalo *et al.* 2007; Swanson-Wagner *et al.* 2009; Shang *et al.* 2015; Liu *et al.* 2012; Zhou *et al.* 2012; Hallin *et al.* 2016; Williams and Williams 2017).

RI genetic reference panels range from inbred lines derived from crosses between two mouse strains to more complex multiparental crosses. The BXD RI panel, derived from two founder strains, C57BL/6J and DBA/2J, has been used to study the impact of genetic variation on susceptibility to IAV infection and map QTL associated with these effects. Boon *et al.* (2009) studied H5N1 infection in females from 66 BXD strains, and Nedelko *et al.* (2012) studied H1N1 infection in 53 BXD strains, with both studies identifying QTL associated with susceptibility to infection. The Collaborative Cross (CC) RI panel is a multiparental population (MPP) descended from eight inbred founder strains (Threadgill *et al.* 2002; Churchill *et al.* 2004), with these founders including representatives from the three major domesticated house mouse subspecies (Yang *et al.* 2011). As such, the CC captures considerably more genetic diversity and, thanks to its breeding structure, this diversity is also more uniformly distributed across the genome, with as many as eight distinct haplotypes segregating at any given locus within the population (Collaborative Cross Consortium 2012; Srivastava *et al.* 2017). The eight CC founder strains have distinct pathogenesis profiles in response to influenza virus (Leist *et al.* 2016), suggesting that the CC RI panel is capable of a broader phenotypic range than would be observed in less complex populations. Indeed, studies using an incompletely inbred, ancestor population of the CC (pre-CC), demonstrated high levels of phenotypic variation across the population and successfully mapped several QTL associated with variation in susceptibility to IAV infection (Ferris *et al.* 2013; Bottomly *et al.* 2012). The CC therefore represents a promising resource for studying how genetically diverse populations respond to IAV infection.

Determining an optimal strategy for how the CC should be used to study the genetic architecture of IAV pathogenesis is nonetheless complicated because (1) the space of possible experimental designs is vast, and (2) information about what types of heritable effects are likely to be present is extremely limited. Regarding (1), with ∼75 CC strains currently available, including all reciprocal F1 hybrids (so called CC-RIXs), there are >5600 potential replicable configurations. Since only a subset of these configurations can be explored within any realistic experiment, any chosen experimental design necessarily targets some types of heritable effects to the exclusion of others. Regarding (2), to date, most *in vivo* studies of IAV pathogenesis have been confined to candidate genes or additive interactions at single loci; studies investigating the broader question of what types of heritability are at play during IAV infection are largely absent.

To rationally design studies of heritable effects in complex populations such as the CC it is therefore helpful to have advance knowledge of which types of heritable effects might be present. One source of such information is phenotype data collected on the multiparental founders and their F1 hybrid offspring, a combination that can be more formally described as an (inbred) diallel. Diallels have a long history in quantitative genetics, having been used originally in plant breeding studies to judge the relative merits of different strain combinations and subsequently for gaining insight into the heritable architecture of a broad range of phenotypes [*e.g.*, references in Christie and Shattuck (1992), Lenarcic *et al.* (2012), and Okoro and Mbajiorgu (2017)], including host–pathogen interactions in, *e.g.*, crickets (Rantala and Roff 2006) and flies (Wayne *et al.* 2011) (see *Statistical Models and Methods* and *Discussion* for connections to other diallel literature).

Here we use a diallel of the CC founders and their reciprocal F1 hybrids (hereafter, a CC founder diallel) to give an overall predictive picture of the range and relative influence of the different types of heritable effects on IAV pathogenesis that are likely to be present in CC founder-derived MPPs, a group that includes not only replicable MPPs such as the CC and the CC-RIX but also irreplicable ones such as the Diversity Outbred (DO) population (Svenson *et al*., 2012). We take advantage of the diallel design’s replicability to measure IAV-induced pathogenesis in a precise way, as the response to an applied treatment defined in terms of postinfection (p.i.) weight-loss differences (deltas) between matched sets of mock and infected individuals (Figure 1). Adapting a recently developed statistical framework for analyzing treatment-response diallels (Crowley *et al.* 2014), we use those deltas to model how pathogenic response to IAV is modulated by parentage, sex, and their interaction, framed in terms of additive genetics, dominance, epistasis, parent-of-origin, and sex-specific versions thereof.

After observing that, following IAV infection, diallel individuals show a broad, continuous distribution of day 4 (D4) p.i. weight loss, we find, through statistical modeling, that the IAV-induced weight loss includes substantial contributions of host additive, epistatic, and sex-specific effects, with much of the heritable variation closely tracking the genotype state implied by the three distinct functional alleles of the previously identified resistance locus *Mx1*. Confirming previous findings, the functional CAST/EiJ (CAST) *Mx1* allele, in contrast with functional NZO/HlLtJ (NZO) and PWK/PhJ (PWK) *Mx1* alleles, provides intermediate levels of protection against H1N1 influenza strains. Unexpectedly, and confirmed through additional modeling, we found that different classes of functional *Mx1* alleles exhibit distinct functional patterns, additive or dominant, when combined with null *Mx1* alleles. Further, illustrating our general rationale, we show that the major strain-specific, *Mx1*-effect patterns are consistent across two CC founder-derived MPPs: the pre-CC, as determined from reanalysis of a previously published data set (Ferris *et al.* 2013), and a previously unpublished 105-line CC-RIX, in which we conduct a limited analysis focused on the *Mx1* locus.

**Figure 1:**
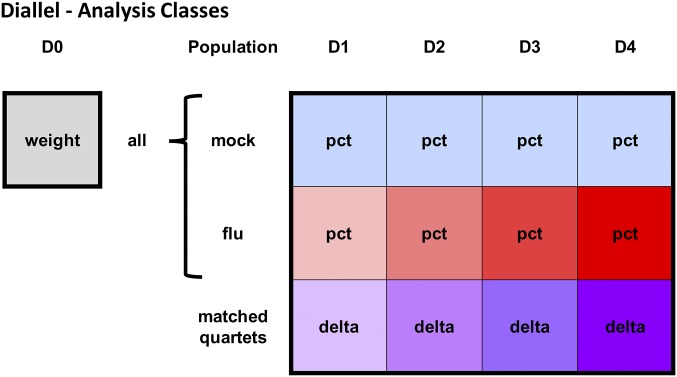
Phenotype and treatment-response classes for analysis of IAV infection in the diallel. Each filled square represents a weight or weight-change phenotype that is modeled independently. The gray square represents the starting body weight in all animals, prior to treatment, at D0 (analyzed with model 1 in Table 2). Light blue squares represent animals that were mock treated and red squares represent animals infected with IAV, with daily weights for each taken from D1 to D4 p.i. (and these were analyzed with model 2 in Table 2). Purple squares represent infection response, the primary quantity of interest, estimated using match quartets of one mock to three infected mice (analyzed with models 3 and 4 in Table 2). Labels within each square indicate phenotypes analyzed, where weight = preinfection body weight, pct = p.i. percent change in starting D0 weight (post), and “delta” = infection response, as described in the *Statistical Models and Methods* section. The coloring increases in saturation from D1 to D4 for the influenza and matched quartet groups to indicate an overall increasing amount of p.i. weight loss over time.

## Experimental Materials and Methods

### CC founder diallel mice

**Figure 2:**
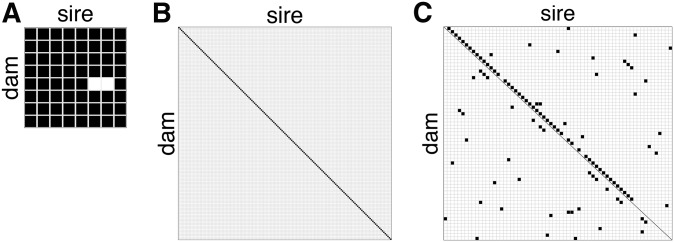
Diagram of breeding strategy for diallel, pre-CC, and CC-RIX. (A) The diallel cross produces inbred (*n* = 8) and F1 (*n* = 54 lines) genotypes from an 8 × 8 cross of inbred strains. (B) The pre-CC is comprised of incompletely inbred (*n* = 155 lines) genotypes from 155 inbreeding funnels. (C) The CC-RIX produces F1 hybrid lines (*n* = 105 lines) from a sparse, round robin-like cross of 65 inbred CC strains.

The inbred and F1 mice used within this study were bred in-house at the University of North Carolina at Chapel Hill (UNC-CH). This colony was directly descended from the subset of animals used to generate the initial CC funnels (Collaborative Cross Consortium 2012) and included mice from the following eight strains at The Jackson Laboratory: A/J (AJ; #000646), C57BL/6J (B6; #000664), 129S1/SvImJ (129; #002448), NOD/ShiLtJ (NOD; #001976), NZO (#002105), CAST (#000928), PWK (#003715), and WSB/EiJ (WSB; #001145). Mice from the UNC-CH colony were then used to generate all 62 possible inbred and (reciprocal) F1 combinations between these eight strains, excluding NZO × CAST and NZO × PWK matings which are nonproductive (Chesler *et al.* 2008) (Figure 2A). This yielded a total of 124 distinct combinations of sex and parentage (hereafter, described as “diallel categories”). Lung tissues were collected from a subset of each of the founder inbred strains in this study, at day 2 (D2) and D4 p.i., and were used for a separate comparative RNA-seq analysis by Xiong *et al.* (2014).

### Mouse infections in the diallel

Mice were weaned at ∼21-d old and housed four per cage, within each diallel category, under standard conditions (12 hr light/dark; food and water *ad libitum*). Of the four mice in a cage, one was randomly assigned to mock and three to influenza infection, as there is no evidence that mice can transmit influenza virus. Each cage was then assigned to a harvest time point: D2 p.i. (*n* = 533 mice), or D4 p.i. (*n* = 510 mice).

At 8–12 wk of age, based on their assignments, mice were anesthetized with isoflurane and inoculated intranasally with 500 plaque-forming units (PFU) of mouse-adapted IAV (H1N1 A/Puerto Rico/8/1934; short name PR8) or with the diluent, phosphobuffered saline (PBS), alone as a mock control. For each inbred strain and F1 cross, about six mice (range: 5–9) of each sex were infected with IAV PR8, and about two mice (range: 2–3) of each sex were mock infected. This gave a total of 1043 mice across 54 experimental batches. Treatment assignment was random: same-sex siblings from the same cage (and therefore batch) were randomly assigned at weaning to mock or infected groups prior to being moved to new cages. The 1043 mice were housed in ∼260 cages (about four mice per cage), with 775 infected mice and 268 mock-infected mice. Body mass was recorded daily. All animal experiments were carried out in compliance with the Guide for the Care and Use of Laboratory Animals (Institute of Laboratory Animal Resources, National Research Council, 1996, https://www.ncbi.nlm.nih.gov/books/NBK232589/). Animal protocols were approved by the Institutional Animal Care and Use Committee of UNC-CH.

### Mouse infections in the pre-CC and CC-RIX

To verify that strain-specific haplotype effects measured in the diallel were consistent with those at the host resistance locus *Mx1*, we sought out CC-related IAV infection data sets for which we could isolate *Mx1* locus-specific effects.

#### Existing data from pre-CC study:

In the QTL mapping study of host response to IAV infection of Ferris *et al.* (2013), 155 female pre-CC mice from as many pre-CC lines were infected with IAV (PR8) at 8–12 wk of age and assayed for p.i. weight loss via daily weights, with phenotypes collected including starting weight (D0) and weight at D4 p.i. (Figure 2B). This study did not include mock-infected mice.

#### CC-RIX study:

In total, 1402 female mice were bred from 105 F1 crosses of CC strains (*i.e.*, 105 CC-RIX lines) (Figure 2C and Supplemental Material Figure S2 in File S7), as part of an ongoing QTL mapping study. These mice were infected at 8–12 wk of age with 5000 PFU IAV (A/California/04/2009; short name CA04), a human 2009 pandemic H1N1 isolate (Itoh *et al.* 2009), and phenotypes were collected, including starting weight (D0) and weight at D7 p.i. CC-RIX were bred under similar conditions to diallel mice. This experiment, whose broader analysis is still ongoing, included both flu-infected and mock-treated mice. However, since the design did not match these to the same exacting degree as the diallel, with mock controls missing entirely for some batch/line combinations, in the current study we consider data from the infected mice only. CC animals used to generate CC-RIX lines were purchased from the Systems Genetics Core at UNC-CH; information about CC strains available for distribution is found at http://csbio.unc.edu/CCstatus/index.py?run=AvailableLines (Morgan and Welsh 2015).

## Statistical Models and Methods

Our statistical analysis of heritable effects in the diallel (hereafter, diallel effects) relies heavily on the BayesDiallel model and approach described by Lenarcic *et al.* (2012) and Crowley *et al.* (2014). BayesDiallel was originally proposed in Lenarcic *et al.* (2012) for diallel analysis of routine, single outcome phenotypes, describing how the mean value of those phenotypes was shifted by changes in parentage and sex. Although in some ways the method was built upon a canon of existing diallel literature (*e.g.*, references in Christie and Shattuck 1992), including more recent work that used random effects (Zhu and Weir 1996; Tsaih *et al.* 2005) and Bayesian hierarchical modeling (Greenberg *et al.* 2010), in other ways it represents a new parameterization and a generalization of many earlier methods [see Lenarcic *et al.* (2012) for explicit connections to those methods]. In Crowley *et al.* (2014), we extended BayesDiallel to treatment-response phenotypes, in particular, to when the modeled outcome is the phenotypic difference between placebo and treated matched pairs; the model in this case describes a causal effect modification, or, in a slight abuse of terminology, a gene-by-treatment (G × T) effect. Herein, that treatment-response approach is extended further to our more complex matching regime of quartets rather than pairs, and with a different imputation procedure to deal with quartets that are incomplete.

This section begins by reviewing the BayesDiallel model for single outcome phenotypes. This is used not only to analyze our primary baseline phenotype, body weight at day 0 (D0 weight), but is also foundational for our subsequent analyses. Then we introduce our definition of infection response based on matched quartets, which gives rise to treatment responses defined for each of four time points (D1, D2, D3, and D4 p.i.), and describe how they are modeled using BayesDiallel. The analysis is then modified further to estimate the impact of haplotype state at the resistance locus *Mx1*, and we describe how the interaction of haplotype pairs at this locus is examined by estimating relative degrees of haplotype additivity and dominance. Finally, we describe an illustrative comparative analysis of the effect of the *Mx1* locus on IAV response in pre-CC and CC-RIX mice.

### Diallel model for single outcome phenotypes

Diallel effects for single outcome phenotypes, that is, phenotypes measured as a single value per mouse, were modeled using the “fulls” model of BayesDiallel (Lenarcic *et al.* 2012; Crowley *et al.* 2014). BayesDiallel is a Bayesian linear mixed model that decomposes phenotypic variation into separate heritable components corresponding to additive genetics, dominance/inbred effects, parent-of-origin (“maternal”), epistasis, and all sex-specific versions thereof. It models the phenotype value $y_{i}$ of mouse *i* as

$$y_{i}=\mu +c_{i}^{\text{T}}\alpha +\sum_{r=1}^{R}u_{i}^{\left(r\right)}+d_{i}^{\text{T}}\beta +\varepsilon_{i}\;,$$ (1)

where *μ* is the intercept, and $\varepsilon_{i}$ is the residual error, normally distributed as $\varepsilon_{i}∼\text{N}\left(0,\sigma^{2}\right),$ with variance $\sigma^{2}.$ The $c_{i}^{\text{T}}\alpha$ term represents the contribution of an arbitrary set of user-specified fixed effect covariates, with predictors encoded in vector $c_{i}$ and fixed effects α; the $\sum_{r=1}^{R}u_{i}^{\left(r\right)}$ term represents the contribution of an arbitrary set of *R* user-defined random effect covariates, which for single outcome phenotypes in this study always includes an effect of experimental batch; and the $d_{i}^{\text{T}}\beta$ term represents the contribution of heritable components of the diallel, written as a linear combination of the diallel effects vector β and diallel category vector $d_{i}.$ Here $d_{i}$ is shorthand for $d_{\left\{jks\right\}\left[i\right]},$ where {jks}[i] denotes *i*’s diallel category, that is, its unique combination of mother strain *j*, father strain *k*, and sex *s*. The diallel category vector $d_{\left\{jks\right\}}$ is defined with the diallel effects β so as to give the linear combination shown in Equation 2, where $a_{j}$ is the additive effect of strain *j* (*e.g.*, the additive effect parameter $a_{\text{AJ}}$ is the expected increase in phenotype on adding one haploid genome of strain AJ); $m_{j}$ is an additional increase in phenotype induced by strain *j* being the mother (parent-of-origin effect); indicator $I_{\left\{X\right\}}$ is 1 if *X* is true and 0 otherwise; $\beta_{\text{inbred}}$ is the overall effect of being inbred; $b_{j}$ is the additional effect of being inbred for strain *j*; $v_{jk}$ is the additional effect of combining strains *j* with *k* regardless of which is the mother (symmetric epistasis); indicator $S_{\left\{X\right\}}$ is 1/2 if *X* is true and −1/2 otherwise; $w_{jk}$ is a deviation from $v_{jk}$ induced by parent-of-origin (asymmetric epistasis); *φ* is the effect of being female rather than male; and $\varphi_{j}^{a}$ is the sex-specific deviation from additive effect $a_{j},$ with other superscripted *φ* terms (*e.g.*, $\varphi^{m}$) defined analogously. Each set of related variables, *e.g.*, the additive effects $a_{1},\ldots ,a_{J}$ for *J* parents, is modeled as a group via a constrained normal distribution, that is, $a_{1},a_{2},\ldots ,a_{J}∼\text{marginally}\;\text{N}\left(0,\tau_{a}^{2}\right),$ but subject to $\sum_{j}a_{j}=0,$ after Crowley *et al.* (2014). The variance of each group, *e.g.*, $\tau_{a}^{2},$ was modeled with a weak inverse gamma prior, $\tau_{a}^{-2}∼\chi^{2}\left(\text{d}.\text{f}.=0.2,\text{mean}=0.2\right),$ with this prior also used for the residual variance $\sigma^{2}.$ The prior for fixed effects, *e.g.*, *μ*, is set to a vague normal distribution, $\mu ∼\text{N}\left(0,10^{3}\right).$ A summary of the diallel effects parameters is given in Table 1. Model fitting proceeded using Markov chain Monte Carlo (MCMC) via Gibbs sampling (algorithm in Lenarcic *et al.* 2012), with results based on samples from $12.5\times 10^{6}$ MCMC iterations (five chains of length 2500, after 500 iterations burn-in). See also the later section *Reporting BayesDiallel results: highest posterior density*, *model inclusion probability*, *variance projection*, *and treatment response variance projections*.

$$\begin{array}{c} d_{\left\{jks\right\}}^{\text{T}}\beta =\underset{\text{additive}}{\underset{︸}{a_{j}+a_{k}}}+\underset{\text{maternal}}{\underset{︸}{m_{j}-m_{k}}}+\underset{\text{inbred}\;\text{penalty}}{\underset{︸}{I_{\left\{j=k\right\}}\left(\beta_{\text{inbred}}+b_{j}\right)}}+\underset{\text{epistasis}}{\underset{︸}{I_{\left\{j\neq k\right\}}\left(v_{jk}+S_{\left\{j<k\right\}}\cdot w_{jk}\right)}}\\ +S_{\left\{s=\text{female}\right\}}\cdot \left[\varphi +\underset{\text{sex}\times \text{additive}}{\underset{︸}{\varphi_{j}^{a}+\varphi_{k}^{a}}}+\underset{\text{sex}\times \text{maternal}}{\underset{︸}{\varphi_{j}^{m}-\varphi_{k}^{m}}}+\underset{\text{sex}\times \text{inbred}\;\text{penalty}}{\underset{︸}{I_{\left\{j=k\right\}}\left(\varphi_{\text{inbred}}+\varphi_{j}^{b}\right)}}+\underset{\text{sex}\times \text{epistasis}}{\underset{︸}{I_{\left\{j\neq k\right\}}\left(\varphi_{jk}^{v}+S_{\left\{j<k\right\}}\cdot \varphi_{jk}^{w}\right)}}\right], \end{array}$$ (2)

**Table 1:** Model parameters, random and fixed (overall), from Equations 1, 2, and 5

Parameter	Color	Description	Type	Levels
*μ* (or *θ*)		Overall mean (or overall infection response)	Fixed	1
*α*		D0 body weight	Fixed	1
$u^{\left(\text{batch}\right)}$		Experimental batch	Random	44 or 52*^a^*
$u^{\left(Mx1\;\text{diplo}\right)}$		*Mx1* diplotype	Random	6
$a_{j}$	Blue	Strain-specific additive	Random	8
$m_{j}$	Green	Strain-specific maternal (parent-of-origin)	Random	8
$\beta_{\text{inbred}}$	Red	Overall inbred penalty	Fixed	1
$b_{j}$	Orange	Strain-specific inbred penalty	Random	8
$v_{jk}$	Purple	Strain pair-specific symmetric epistasis	Random	28
$w_{jk}$	Brown	Strain pair-specific asymmetric epistasis (parent-of-origin)	Random	28
*φ*	Gray	Overall female	Fixed	1
$\varphi_{j}^{a}$	Light blue	Sex-by-strain-specific additive	Random	8
$\varphi_{j}^{m}$	Light green	Sex-by-strain-specific maternal (parent-of-origin)	Random	8
$\varphi_{\text{inbred}}$	Pink	Overall female inbred	Fixed	1
$\varphi_{j}^{b}$	Light orange	Sex-by-strain-specific inbred penalty	Random	8
$\varphi_{jk}^{v}$	Lavender	Sex-by-strain pair-specific symmetric epistasis	Random	28
$\varphi_{jk}^{w}$	Tan	Sex-by-strain pair-specific asymmetric epistasis (parent-of-origin)	Random	28

a Random effect levels for $u^{\left(\text{batch}\right)}$ differ according to the number of experimental batches within each phenotype being modeled: 52 levels for D0, D1pct, D2pct, D1delta, D2delta; and 44 levels for D3pct, D4pct, D3delta, and D4delta. In the text, *h* is used to indicate the level of batch for a given individual or quartet.

### Modeling infection response as mock-corrected percent change in body weight p.i.

A standard measure used to assess pathogenesis in IAV-infected mice is weight loss. Weight loss correlates with several host and viral factors, including viral load, immune response phenotypes, and lung histopathology (Ferris *et al.* 2013; Leist *et al.* 2016); as such, it provides a simple, noninvasive measure of infection pathology that can be assessed for a large number of mice. We measured the percentage change in body weight relative to D0,

$${\text{pct}}_{i}^{\text{day}\left[\text{group}\right]}=100\times {\text{weight}}_{i}^{\text{day}\left[\text{group}\right]}/{\text{weight}}_{i}^{\text{D}0\left[\text{group}\right]},$$ (3)

for mouse *i* on day∈{D1,D2,D3,D4} in group∈{flu,mock}, where, *e.g.*, ${\text{weight}}_{i}^{\text{D}4\left[\text{flu}\right]}$ and ${\text{weight}}_{i}^{\text{D}0\left[\text{flu}\right]}$ are the body weights for IAV-infected mouse *i* at D4 and at D0, respectively. These measures, which we describe as single outcome phenotypes, were analyzed using BayesDiallel as above (Table 2), but they were not the main focus of our study. Our main focus was a derived measure, IAV-infection response, defined next.

**Table 2:** Models used for each analysis in this study

Model Number	Model*^a^*		Phenotype(s)	Unit	Variance Parameters
1	$y_{i}=\mu +u_{i}^{\left(\text{batch}\right)}+$	$d_{i}^{\text{T}}\beta +\varepsilon_{i}$	Pre*^b^*	Individuals	12*^c^*
2	$y_{i}=\mu +u_{i}^{\left(\text{batch}\right)}+c_{i}^{\left(\text{D}0\right)}\alpha +$	$d_{i}^{\text{T}}\beta +\varepsilon_{i}$	Post*^d^*	Individuals	12*^c^*
3	${\mathrm{delta}}_{q}=\theta +u_{q}^{\left(\text{batch}\right)}+c_{q}^{\left(\text{D}0\right)}\alpha +$	$d_{q}^{\text{T}}\beta +\varepsilon_{q}$	delta*^e^*	Quartets	12*^c^*
4	${\mathrm{delta}}_{q}=\theta +u_{q}^{\left(\text{batch}\right)}+c_{q}^{\left(\text{D}0\right)}\alpha +u_{q}^{\left(Mx1\;\text{diplo}\right)}+$	$d_{q}^{\text{T}}\beta +\varepsilon_{q}$	delta*^e^*	Quartets	13*^f^*

a See study design in Figure 1 for overview of analyses. See Table 1 and *Statistical Models and Methods* for parameter and phenotype definitions.

b D0 [all].

c This count includes $\tau_{\text{batch}}^{2},$ {$\tau_{a}^{2},$ $\tau_{m}^{2},$ $\tau_{b}^{2},$ $\tau_{v}^{2},$ $\tau_{w}^{2}$}, {$\tau_{\varphi^{a}}^{2},$ $\tau_{\varphi^{m}}^{2},$ $\tau_{\varphi^{b}}^{2},$ $\tau_{\varphi^{v}}^{2},$ $\tau_{\varphi^{w}}^{2}$}, and $\sigma^{2}.$

d D1pct, D2pct, D3pct, D4pct [mock] and D1pct, D2pct, D3pct, D4pct [flu].

e D1delta, D2delta, D3delta, and D4delta.

f This count includes $\tau_{Mx1\;\text{diplo}}^{2}$ and parameters in *c*.

In defining IAV-infection response, we note that from a causal inference perspective (described more fully in Appendix A) weight loss in an IAV-infected mouse (*e.g.*, ${\text{pct}}_{i}^{\text{D}4\left[\text{flu}\right]}$) reflects two confounded processes: weight loss due to IAV-induced pathogenesis, and weight loss due to other aspects of the experimental procedure. To obtain an unconfounded estimate of weight loss due to IAV-induced pathogenesis alone, we defined IAV-infection response as the difference between weight loss in mice subject to infection by IAV and those subject to mock. Specifically, since in our experimental design we match one mock mouse to three infected—this reflecting our expectation that phenotypes from infected mice will be more variable and will thus need more replicates for comparable precision—infection response was defined in terms of “matched quartets,” q=1,…,Q, where each matched quartet *q* comprised four mice of the same diallel category from the same experimental batch, with the first three mice, q[1], q[2] and q[3], being IAV infected and the last mouse, q[4], receiving mock treatment. Infection response at a given day for quartet *q* was thus defined as a delta,

$${\text{delta}}_{q}^{\text{day}}=\frac{1}{3}\sum_{f=1}^{3}{\text{pct}}_{q\left[f\right]}^{\text{day}\;\left[\text{flu}\right]}-{\text{pct}}_{q\left[4\right]}^{\text{day}\;\left[\text{mock}\right]}\;,$$ (4)

following the more general definition in Equation A2 in Appendix A.

Diallel effects on infection response were then modeled using BayesDiallel in manner analogous to the single outcome case in Equation 1, as

$${\text{delta}}_{q}^{\text{day}}=\theta +c_{q}^{\text{T}}\alpha +\sum_{r=1}^{R}u_{q}^{\left(r\right)}+d_{q}^{\text{T}}\beta +\varepsilon_{q}\;,$$ (5)

where now the unit of observation is the matched quartet *q* rather than the individual *i* and where, for example, $d_{q}$ is shorthand for $d_{\left\{jk,s\right\}\;\left[q\right]},$ the diallel category appropriate for *q*. The shift to modeling treatment response does, however, change how the parameters are interpreted. The intercept in the above formula, relabeled as *θ*, now acquires a special meaning, representing an overall causal effect due to infection, and the diallel effects in β now describe how that causal effect is modified by parentage, sex, and their interaction. For example, the additive effect parameter $a_{\text{AJ}}$ is the expected increase in infection response on adding one haploid genome of strain AJ. Regarding covariates, as for the single outcome phenotypes, this model included a random effect of batch and, to reduce potential dependence between the delta’s and baseline body weight, we also included a fixed effect covariate for the quartet mean D0 weight (*i.e.*, $\text{D}0_{q}=\sum_{f=1}^{4}{\text{weight}}_{q\left[f\right]}^{\text{D}0}/4$) in $c_{q}$ (Table 2).

Although our experimental design stipulated even multiples of four mice per diallel category, practical constraints on animal breeding and availability meant that in some cases this number was three or five, such that some quartets had either missing infecteds or surplus mocks. To ensure the definition of delta in Equation 4 remained consistent, and in particular that delta’s from different quartets had comparable precision, the diallel analysis was performed on M=1000 imputed versions of the data, with each imputed data set being comprised of exact quartets in which missing phenotypes had been filled using stochastic regression imputation and surplus mocks had been (randomly) deleted (details in Appendix B). On each imputed data set we collected 125 MCMC samples from 12,500 total time steps (*i.e.*, by recording values at every 100th time step); results were based on the aggregate of these samples from the *M* imputed data sets (*i.e.*, on 125,000 MCMC samples in total).

### Reporting BayesDiallel results: highest posterior density, model inclusion probability, variance projection, and treatment response variance projections

Point and interval estimates of individual diallel effects, *e.g.*, additive effect $a_{\text{AJ}},$ are reported as posterior means and 95% highest posterior density (HPD) intervals. The overall contribution of a particular inheritance group is reported in two ways: as a variance projection (VarP), *e.g.*, VarP[a] for the contribution of additive effects to a phenotype or treatment response VarPs (TreVarPs), *e.g.*, TreVarP[a] for the contribution of additive effects to an infection response; and as a model inclusion probability (MIP), *e.g.*, MIP[a] for the probability of additive effects being included in the model.

The VarP is a heritability-like measure that predicts how much of the total phenotypic sum of squares would be explained by each component in a new, completely balanced diallel. Unlike traditional heritability, it is calculated based on the effects, β, rather than the variance components, $\tau_{a}^{2},\ldots ,\tau_{w}^{2},\sigma^{2},$ and as such benefits not only from greater interpretability but also from the stability and accuracy provided by hierarchical shrinkage (as detailed in Crowley *et al.* 2014). Since the VarP is a function of the posterior predictive distribution and calculated at each iteration of the MCMC, it is reported via Bayesian posterior summaries, specifically, the posterior median and the 95% HPD interval. The VarPs for infection response phenotypes are, following Crowley *et al.* (2014), given the special name of TreVarPs to acknowledge their more delicate interpretation.

The MIP reflects a different type of inference: rather than being a function of the parameters estimated in the full, sexed BayesDiallel model of Equations 1 and 2, it describes the results of model selection, that is, an assessment of which diallel categories could be excluded without a substantial loss in fit. As in Crowley *et al.* (2014), we use the exclusionary Gibbs group sampler of Lenarcic *et al.* (2012). Each diallel category is set to have a prior inclusion probability of 0.5, reflecting a prior opinion that inclusion and exclusion are equally likely. This prior is then updated by the phenotype data and the model selection procedure to give a (posterior) MIP. MIPs are interpreted following the conventions in Crowley *et al.* (2014): MIPs in the range (0.25, 0.75) indicate that the data does not provide sufficient evidence to make an informed decision about exclusion or inclusion; MIPs within (0.05, 0.25] or [0.75, 0.95) represent positive evidence for exclusion or inclusion respectively; (0.01, 0.05] or [0.95, 0.99) represent strong evidence; and [0, 0.01] or [0.99, 1] represent strong to decisive evidence. These conventions are based on those proposed by Kass and Raftery (1995) for Bayes factors, which are connected to MIPs by the relation

$$\text{Bayes}\;\text{factor}=\frac{\text{MIP}}{1-\text{MIP}}\times \frac{1-{\text{MIP}}_{0}}{{\text{MIP}}_{0}},$$

where ${\text{MIP}}_{0}$ is the prior inclusion probability, and where the above simplifies to MIP/(1−MIP) in our case of ${\text{MIP}}_{0}=0.5.$

### Estimating Mx1 effects in the diallel

The critical host resistance factor (*Mx1*) has been shown to drive IAV resistance in the CC founder strains and has been mapped in the pre-CC (Ferris *et al.* 2013). *Mx1* was previously described as having three major, naturally occurring functional classes of resistance to influenza H1N1 arising from the subspecies *Mus musculus domesticus* (hereafter, *dom*; members include AJ, B6, 129, NOD, and WSB), *M. musculus castaneus* (*cast*; CAST), and *M. musculus musculus* (*mus*; PWK and NZO), of which *domesticus* is considered to be null whereas *musculus* and *castaneus* are protective. (Note that *domesticus Mx1* in the CC founder strains is comprised of two unique null alleles, and that the subspecific *Mx1* alleles observed in the CC may not be representative of the those segregating in the wild.) To estimate the contribution of *Mx1* haplotypes as discernible in the diallel, and thereby also estimate the extent of heritable effects that remain after *Mx1* is controlled for, we define the following haplotype combinations (diplotypes) as six levels of the random effect, $u^{\left(Mx1\;\text{diplo}\right)}:$ {*dom* × *dom*}, {*dom* × *cast*}, {*cast* × *cast*}, {*cast* × *mus*}, {*mus* × *dom*}, and {*mus* × *mus*}; we then repeat our diallel analysis with this effect included (model 4 in Table 2).

#### Estimating a dominance index for Mx1 alleles:

Dominance is typically defined in the context of bialleles, but since in this population *Mx1* has a multiallelic series, we define dominance of *Mx1* between allele pairs. Following Kacser and Burns (1981), which is built on the work of Wright (1934), we define the “dominance index” for a wild-type (wt) against a mutant (mut) allele as

$$D^{\left(\text{wt};\;\text{mut}\right)}=\frac{u^{\left(\text{wt wt}\right)}-u^{\left(\text{wt mut}\right)}}{u^{\left(\text{wt wt}\right)}-u^{\left(\text{mut mut}\right)}},$$ (6)

where values for D are close to −0.5 when the effect of the wt is overdominant to the mut (the effect of the mut is underrecessive), 0 when the effect of the wt is completely dominant to the mut (the effect of the mut is recessive), close to 0.5 when the effect of the wt is additive (not dominant, or incompletely dominant) to the mut, close to 1 when the effect of the wt is recessive (the effect of the mut is dominant), and close to 1.5 when the effect is underrecessive (the effect of the mut is overdominant). Overdominance is given by values of D that are much less than zero and underdominance by values that are much greater than one. This definition is used to define dominance indices $u^{\left(cast;\;dom\right)}$ and $u^{\left(mus;\;dom\right)},$ describing the degree of dominance of the protective alleles *castaneus* and *musculus*, respectively, against the null allelle *domesticus*. To assess the degree to which *castaneus* and *musculus* differ in their relation to *domesticus*, we further define a “dominance difference index,”

$$DD^{\left(mus-cast;\;dom\right)}=D^{\left(mus;\;dom\right)}-D^{\left(cast;\;dom\right)}\;,$$ (7)

where negative values indicate that *musculus* has more of a dominance-based relationship to *domesticus* than does *castaneus*, positive values indicate the converse, and zero indicates that the relationships of *castaneus* and *musculus* to *domesticus* show dominance equally.

When the BayesDiallel model includes *Mx1* effects, the aforementioned dominance index and dominance difference index are both functionals of the posterior; posterior samples of these indices were therefore obtained by simply applying Equations 6 and 7 to the sampled *Mx1* effects at each time step of the MCMC.

The Kacser and Burns (1981) dominance index is a simple reparameterization of the degree of dominance parameter, $a_{\text{CR}},$ defined by Comstock and Robinson (1948) and used by Gardner and Lonnquist (1959). In the Comstock–Robinson model, the mean-centered phenotypes are coded as (translating from our model above): $u^{\left(\text{wt};\;\text{wt}\right)}=w,$ $u^{\left(\text{wt};\;\text{mut}\right)}=aw,$ and $u^{\left(\text{mut};\;\text{mut}\right)}=-w.$ This gives the relation $D^{\left(\text{wt};\;\text{mut}\right)}=\left(1-a_{\text{CR}}\right)/2$ or equivalently, $a_{\text{CR}}=1-2D^{\left(\text{wt};\;\text{mut}\right)}.$ This alternate dominance parameterization is explored further using BayesDiallel in Turner *et al.* (2017).

### Estimating haplotype effects at the Mx1 locus in the pre-CC and CC-RIX

The additive effect parameters estimated in the diallel do not precisely distinguish the effects at the *Mx1* locus because they are confounded with any potential genome-wide effects that follow the same pattern of strain classification. An unconfounded estimate of haplotype effects at *Mx1* requires a population in which the remainder of the genome is randomized, *e.g.*, by recombination. To this end, we make use of two related data sets on IAV-induced weight loss in two CC-derived MPPs: IAV (PR8) infection in the pre-CC and IAV (CA04) infection in a set of CC-RIX lines. These two studies, described in more detail below, were in other respects less rigorous than our diallel: the experimental measurement of the infection response was based on infected mice only with no mocks in the pre-CC, and although mocks were collected in the CC-RIX, their relative sparsity (200–300 mocks to >1400 infecteds) complicates analysis based on matching alternate treatment groups; the experimental batching was subject to a less exacting degree of randomization across genetically distinct categories; the available combinations of *Mx1* diplotypes are limited mostly to homozygotes in the pre-CC, and incompletely and unevenly sampled in the CC-RIX; and the *Mx1* diplotype state for each line is known only probabilistically, having been inferred by hidden Markov models (HMMs) applied to genotyping data. Nonetheless, if effects at the *Mx1* locus were largely independent of those elsewhere in the genome, we might expect that *Mx1* effects in the pre-CC and CC-RIX would be broadly consistent with those in the diallel.

Estimation of haplotype effects at the *Mx1* locus was performed using the Diploffect model (Zhang *et al.* 2014), a Bayesian hierarchical model that estimates effects of diplotype substitutions at a specified QTL when the diplotype states themselves are known only probabilistically. The effects estimated by Diploffect are analogous to those estimated by BayesDiallel: phenotype $y_{i}$ of mouse *i* is modeled as

$$y_{i}=\mu +c_{i}^{\text{T}}\alpha +\sum_{r=1}^{R}u_{i}^{\left(r\right)}+{\text{dip}}_{i}^{\text{T}}\beta +\varepsilon_{i}\;,$$ (8)

where ${\text{dip}}_{i}$ is a vector representing the diplotype state of mouse *i* at the QTL and is shorthand for ${\text{dip}}_{\left\{jk\right\}\left[i\right]},$ where {jk}[i] denotes *i*’s diplotype state comprised of haplotypes from CC founder strains *j* and *k*, β are the corresponding effects, and all other variables are as in Equation 1. The diplotype vector ${\text{dip}}_{\left\{jk\right\}}$ is defined with β so as to give the linear predictor

$${\text{dip}}_{\left\{jk\right\}}^{\text{T}}\beta =a_{j}+a_{k}+I_{\left\{j\neq k\right\}}\gamma_{jk}\;,$$ (9)

where $a_{j}$ and $a_{k}$ are additive (haplotype) effects modeled as $a_{j}∼\text{N}\left(0,\tau_{\text{add}}^{2}\right),$ broadly equivalent to the additive effects in BayesDiallel’s Equation 2; and $\gamma_{jk}∼\text{N}\left(0,\tau_{\text{dom}}^{2}\right)$ are dominance deviations, which are the converse to BayesDiallel’s inbred parameters. Dominance deviations are expected to be poorly informed when heterozygotes are sparsely represented, as in the CC-RIX and in particular the largely inbred pre-CC, but are nonetheless included to stabilize inference of additive effects. For numerical stability, phenotypes were first centered and scaled to unit variance, and variance parameters ($\sigma^{2}$ or $\tau_{\text{effect}}^{2},$ where effect is add, dom, or r∈R) were given mildly informative priors of the form $\tau_{\text{effect}}^{-2}∼\text{Ga}\left(1,1\right).$ Estimation proceeded by importance sampling (the DF.IS and DF.IS.kinship methods in Zhang *et al.* 2014) using integrated nested Laplace approximations (INLA; Martins *et al.* 2013), with 100 importance samples taken, and parameter estimates for additive effects are reported as posterior means, posterior medians, and HPD intervals.

#### Pre-CC study:

In the study of Ferris *et al.* (2013), IAV-infection response was measured on 155 mice from as many pre-CC lines as weight loss following infection with IAV (PR8 variant, as for the diallel). QTL mapping of D4 p.i. weight loss, equivalent to ${\text{pct}}_{i}^{\text{D}4}$ in the diallel study, identified a QTL, *HrI1*, containing the *Mx1* gene, with peak marker JAX00072951 (chr16:98,148,641; Mouse Diversity Array of Yang *et al.* 2009). We estimated haplotype effects at this peak marker using Diploffect (Zhang *et al.* 2014), applied to the phenotype and the original HMM probabilities of Ferris *et al.* (2013), with the model including a fixed effect covariate for D0 weight.

#### CC-RIX study:

For the CC-RIX study of infection response to IAV (CA04 strain), we calculated weight loss values for all 1402 infected mice at D7 p.i. (analogous to a ${\text{pct}}_{i}^{\text{D}7}$ measure), and for all 105 CC-RIX lines obtained diplotype probabilities at marker UNC27478095 (16:97,591,482; MegaMUGA array, described in Morgan *et al.* 2016) from the Inbred Strain Variant database (ISVdb; Oreper *et al.* 2017). Haplotype effects were then estimated by Diploffect applied to debatched CC-RIX line means as follows. First, we fit a linear mixed model (by REML using the R package lme4 of Bates *et al.* 2015) to the individual-level phenotypes (*n* = 1402) with fixed effects of D0 weight and laboratory (two levels), and random effects of mating (107 levels: 105 RIXs + 2 additional levels distinguishing minor breeding differences, when CC010 and CC042 strains were rederived from breeder females into a new facility) and infection date (59 levels). The residuals of this model were then averaged over the $n_{i}$ mice of each CC-RIX line *i* and used as the response $y_{i}$ in Equation 8 with precision weighting $\varepsilon_{i}∼\text{N}\left(0,\sigma^{2}/n_{i}\right)$ and a between-line polygenic random effect $u∼\text{N}\left(0,\;G\tau_{G}^{2}\right),$ where the 105×105 genetic relationship matrix G was calculated between all CC-RIX pairs based on the founder haplotype probabilities (dosages) at each locus, according to the method described in Gatti *et al.* (2014).

### Data availability

Analyses were conducted in the statistical programming language R (R Core Team 2017). In addition to R packages cited above, we used the packages BayesDiallel (Lenarcic *et al.* 2012) and Diploffect.INLA (Zhang *et al.* 2014). The data, analysis software, and scripts are available on the flu-diallel repository on GitHub at https://github.com/mauriziopaul/flu-diallel. A static version is posted as a public, open-access Zenodo repository at http://dx.doi.org/10.5281/zenodo.293015. Phenotype data from the diallel and CC-RIX animals used in this study will be available on the Mouse Phenome Database (Grubb *et al.* 2014) at https://phenome.jax.org with persistent identifier RRID:SCR_003212.

File S1 contains an account of the supplemental files which can be used to reproduce our analysis. File S2 contains the software packages used for this analysis. File S3 contains the diallel data file, and File S4, File S5, and File S6 contain the data analysis files required for analyzing the diallel, pre-CC, and CC-RIX, respectively. File S7 contains supplemental figures, tables, and an algorithm. After unzipping, the files FluDiData.csv, Flu-pre-CC-data.csv, and Flu-CC-RIX-data.csv contain raw phenotypes, cross (or line, strain), and mouse ID information from the three mouse populations used in this study. The script files MIMQ*.sh are used in bash to call R scripts to run the BayesDiallel analysis on diallel phenotypes. The script files main_analysis*.R are used with Diploffect to run Diploffect analysis on the pre-CC and CC-RIX phenotypes. Additional *.RData, *.pl, *.alleles, and *.csv files are uploaded which contain settings, genotypes, and founder haplotype probabilities used by the scripts.

## Results

Mice from the eight inbred founder strains of the CC were used to generate a near-complete 8×8 diallel. This study used offspring (*n* = 1043) of both sexes (519 females and 524 males) representing 62 of the 64 crosses (Figure S1 in File S7), including all inbred combinations (*n* = 129) and all F1 hybrids (*n* = 914) except NZO × CAST and NZO × PWK. Within each diallel category—defined as the combination of sex and (reciprocal) parentage—and in each experimental batch, mice were randomly assigned at weaning to infection or mock groups in a ratio of 3:1; complete sets of three infected with one mock were described as matched quartets. Mice in the infected group were inoculated with IAV PR8, and in the mock group with PBS. For each mouse, body weight was measured prior to infection (D0 or baseline weight), and at days 1–4 p.i. (D1, D2, D3, D4). D0 weight is reported in grams whereas p.i. weight is hereafter reported as a percentage of D0 weight, *e.g.*, D4pct. Not all mice survived the protocol: one infected mouse died after D3 weights were taken and one mouse died from anesthesia on D0.

### F1 hybrids of the CC founders show a wide range of phenotypic outcomes

The CC founders include five strains we have previously characterized as susceptible to IAV-induced pathology (AJ, B6, 129, NOD, and WSB), two strains as resistant (NZO and PWK), and one (CAST) that exhibits a distinct intermediate weight loss phenotype (Ferris *et al.* 2013). Results for the inbred founders measured in our diallel replicate those earlier findings, and the p.i. weight loss among the infected F1 hybrids spanned the range of phenotypes observed in the founders (Figure S3 in File S7), consistent with the notion of IAV-induced weight loss being a complex trait with contributions from multiple loci.

### Diallel effects on baseline mouse weight strongly replicate previous CC founder diallel studies

The effects of parentage and sex on D0 weight were estimated using BayesDiallel. Described further in *Statistical Models and Methods*, BayesDiallel decomposes the heritable effects observable in the diallel into 160 parameters (diallel effects) grouped into 13 distinct heritability classes. In sketch form, it models the average phenotype of mice of sex *s* bred from mother of strain *j* and father of strain *k* as

$$\text{ave}.{\text{phenotype}}_{jks}=\begin{array}{c} \text{overall}\;\text{mean}\\ \&\;\mathrm{covariates} \end{array}+\underset{\text{diallel}\;\text{effects}}{\underset{︸}{a_{j}+a_{k}+{\text{inbred}}_{j}+{\text{other}}_{jks}}},$$

where covariates always includes experimental batch, $a_{j}$ and $a_{k}$ are the additive effects of the two parents, ${\text{inbred}}_{j}$ is an additional effect included only when j=k, and ${\text{other}}_{jks}$ models the effects of further nuances of sex and parentage as deviations from this base model (listed in *Statistical Models and Methods* and Table 1).

Diallel effects estimated for D0 weight are reported in Figure S6A in File S7 as 95% HPD intervals for each parameter, and two summary measures, VarPs and MIPs, for each of the 13 heritability classes are given in Figure S6, B and C, in File S7. Briefly, VarPs (Figure S6C in File S7) report the contribution of the effect group as the proportion of the total phenotypic variance, whereas MIPs (Figure S6B in File S7) assess the strength of support for whether an effect group should be included at all, with probabilities near 1 providing stronger support for inclusion, probabilities near 0 supporting exclusion, and probabilities near 0.5 reflecting a lack of information either way.

The pattern of effects for D0 weight was strikingly similar to that seen for baseline body weight in two previous diallels of the CC founders (Lenarcic *et al.* 2012; Crowley *et al.* 2014), despite those earlier studies being independent experiments with no particular attempt made to align experimental protocols, and included substantial additive effects, strain-specific parent-of-origin effects, signals of epistasis, and sex-specific versions thereof. For example, we largely replicated the pattern of inbred, additive, and maternal effects observed in both Lenarcic *et al.* (2012) and Crowley *et al.* (2014), and also found a higher-order, sex-specific PWK × CAST symmetric epistatic effect in Lenarcic *et al.* (2012). We also observed some new epistatic and sex-specific epistatic effects largely due to increased power from a larger sample size.

### Diallel effects on IAV-infection response

Infection response was defined as the percentage change in body weight induced by IAV infection, with more negative values indicating more severe pathology. This was calculated at each time point—D1, D2, D3, and D4 p.i.—as the difference between matched infected and mock mice, yielding a single infection response number (a delta, *e.g.*, D4delta) for each matched quartet (three infected mice and one mock). The effects of parentage and sex on infection response were then analyzed for each time point separately using BayesDiallel as above, with an additional covariate of D0 weight (see *Statistical Models and Methods* for details). Although results are provided in File S7 for all time points, we will focus on those for D4 p.i. since this showed the greatest difference between infected and mock.

### IAV infection causes weight loss through D4 p.i., with greater susceptibility in females

**Figure 3:**
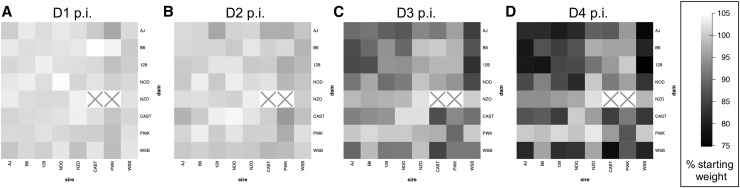
Influenza-induced weight loss in an 8 × 8 diallel cross of mice, through 4 d p.i. Mean weight change, as % D0 weight, is shown at (A) D1, (B) D2, (C) D3, and (D) D4 p.i. with 500 pfu IAV (PR8) in male and female inbreds and F1 hybrids of CC founder strains (*n* = 774 for D1 and D2, *n* = 382 for D3, and *n* = 381 for D4). Results from mock-infected mice not shown. Squares with a gray “X” indicate matings that do not produce offspring.

**Figure 4:**
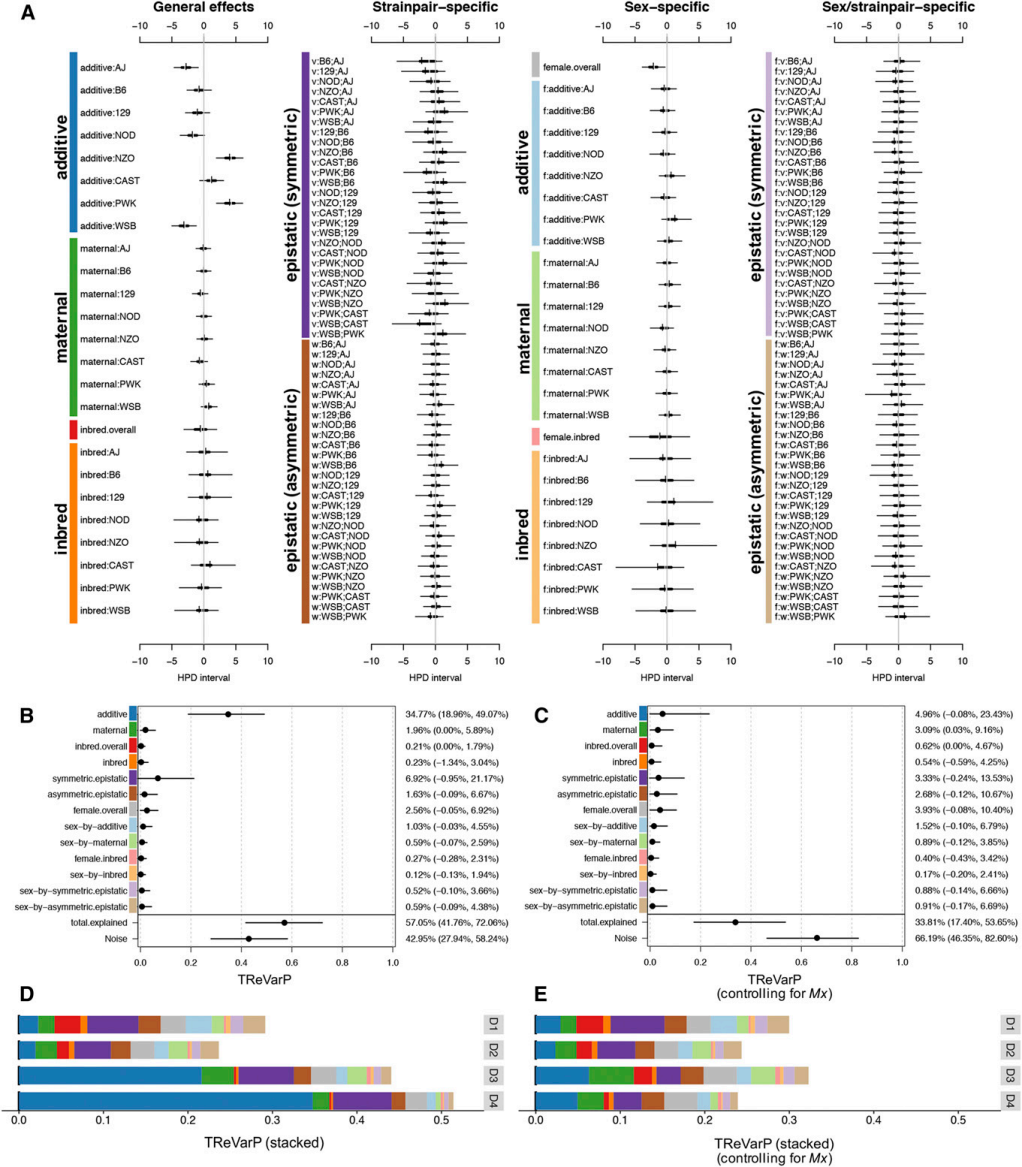
Diallel effects on host weight IAV-infection response, before and after accounting for *Mx1* haplotypes. (A) Effect estimates for additive, maternal, inbred, and epistatic effects, including sex-specific effects, are presented as HPD intervals across 163 individual effects categories for IAV-induced weight change at D4 p.i. (phenotype D4delta). HPDs are given for each parameter, including 95% (thin line) and 50% (thick line) intervals, and median (white break) and mean (black vertical line). Parameters are labeled according to the methods. Symmetric epistatic, asymmetric epistatic, and sex-specific parameters are indicated by “v:”, “w:”, and “f:”, respectively. The overall treatment effect (data not shown), *θ*, is −8.85% (−9.92, −7.78%). (B and C) TReVarPs, a generalization of heritability for diallel effects classes, at D4 are shown for three fixed (overall) effects, five random effects classes, and five corresponding sex-specific random effects classes (posterior median and 95% HPD intervals) before (B) and (C) after accounting for diplotypes at the host influenza resistance locus, *Mx1*. (D and E) TReVarPs before and after *Mx1* for all four p.i. time points.

IAV infection in the diallel induced an overall mean change in body weight (*i.e.*, overall infection response *θ* in Equation 5 and Table 1) of −0.13% (95% HPD interval: −0.48, 0.22; MIP = 1) on D1 p.i., −0.83% (−1.33, −0.32%; MIP = 1) on D2 p.i., −5.60% (−6.47, −4.73%; MIP = 1) on D3, and −8.85% (−9.92, −7.78%; MIP = 1) on D4 (Table S3 in File S7; see also progression in Figure 3). Consistent with previous mouse studies of sex effects on infection (Lorenzo *et al.* 2011; Robinson *et al.* 2011), females given the same dose of virus as male mice had increased weight loss: a negative effect of female sex was estimated at all four time points p.i., gradually increasing in magnitude from −0.89% (−1.45, −0.36%) at D1 p.i. to −2.11% (−3.87, −0.30%) at D4 p.i. (Figure 4), suggesting that enhanced susceptibility in females may occur at least as early as D1 p.i. Although all mice received the same dose of virus regardless of starting body weight, heavier mice experienced a transient increase in percent weight loss at D2 p.i. compared with lighter mice: the D0 weight effect (*α* in Equation 5) on the infection response at D2 p.i. was −0.31% (−0.52, −0.09%), such that for every 10 g of starting weight beyond 0 g, an additional ∼3.1% weight was lost on D2; however, this effect disappeared by D3 p.i. No other significant effects of starting weight on IAV-induced weight loss were detected at other time points, indicating that heavier mice were infected at least as effectively as lighter mice, and that starting body weight does not in general confound our exploration of strain- and cross-specific effects.

#### Diallel effects on infection response reflect mostly additive genetics, consistent with differences in Mx1 haplotype:

Infection response in our diallel was strongly driven by additive effects. On D3 p.i., enhanced susceptibility to weight loss in infected animals was affected the most by contributions from strain AJ, −2.17% (−3.72, −0.61%), and enhanced resistance from contributions of NZO, 2.54% (0.72, 4.27%), and PWK, 1.70% (0.12, 3.23%), strains. On D4 p.i., enhanced susceptibility was greatest from AJ, −2.77% (−4.66, −0.86%), and WSB, −3.09% (−5.01, −1.18%), with enhanced resistance greatest from NZO, 4.07% (1.95, 6.12%), and PWK, 4.06% (1.97, 6.08%) (Figure 4A). In terms of its additive effect, CAST was more resistant than the *Mx1*-null strains (AJ, B6, 129, NOD, and WSB) but about half as resistant as the *Mx1*-functional strains (NZO and PWK), consistent with it conferring intermediate protection in the heterozygote state.

To summarize these effects: for each dose of AJ or WSB genomes inherited from a parent, ∼2–3% of additional starting body weight is lost p.i., indicating enhanced susceptibility compared with the overall mean weight loss; for each NZO and PWK genome inherited, ∼4% more of starting body weight is retained p.i., compared with the mean treatment effect, indicating enhanced resistance.

Diallel effects explained over half of the total variance of infection response at D4, with a treatment-response VarP for all effect groups collectively of 57% (TReVarP[all] = 0.571; 0.418, 0.721). The variance explained by additive effects only, which is related to the narrow-sense heritability, was estimated as 34.8% (TReVarP[a] = 0.348; 0.190, 0.491), and also detected were potential additional contributions of epistasis (TReVarP[v] = 0.069; −0.001, 0.212) and maternal effects (TReVarP[m] = 0.020; 0.000, 0.059) (Figure 4, B and D and Table S1 in File S7).

#### Evidence for additive, inbred, epistatic, and parent-of-origin effects mounts as disease progresses:

The relevance of diallel effects to infection response became more marked with time (Figures S7–S10 and Table S3 in File S7). At D1 and D2 p.i., model inclusion probabilities gave strong support only to an overall infection response, with no evidence of this effect being modified by sex or parentage (Figures S7 and S8 in File S7). At D3 p.i., however, we found positive to strong evidence of additive (MIP[a] = 0.978), inbred (MIP[b] = 0.958), and asymmetric epistatic (MIP[w] = 0.820; *i.e.*, parent-of-origin epistatic) effects (Figure S9 in File S7). By D4 p.i., support for additive (MIP[a] = 0.998) and inbred (MIP[b] = 0.999) effects had become decisive (see *Statistical Models and Methods* for MIP interpretation) and there was strong support for both symmetric epistatic (MIP[v] = 0.960) and asymmetric epistatic (MIP[w] = 0.966) effects (Figure S10 and Table S3 in File S7).

### Modeling effects consistent with Mx1 haplotype

To help distinguish diallel effects that are consistent with the subspecies haplotype of the resistance factor *Mx1* (hereafter, *Mx1* effects), we incorporated the *Mx1* subtype explicitly into the model as a genotype covariate with three alleles, one for each subspecies branch: *domesticus* (AJ, B6, 129, NOD, and WSB), *castaneus* (CAST), and *musculus* (NZO and PWK).

#### Mx1 effects are increasingly evident with disease progression and explain ∼40% of the diallel effects at D4 p.i.:

In keeping with the increased support seen for diallel effects over time, evidence for a nonzero *Mx1* effect increases from positive evidence of exclusion on D1 (MIP = 0.035), to no evidence for inclusion or exclusion on D2 (MIP = 0.552), to decisive evidence for inclusion on D3 (MIP = 1.000) and D4 (MIP = 1.000) (Figures S11–S14 in File S7); a comparable level of support for inclusion in the model was seen only for effects of overall treatment and batch. After controlling for *Mx1*, the variance explained by diallel effects at D4 was substantially reduced, from 57 to 33.8% (TReVarP[all|Mx1] = 0.338; 0.174, 0.537) (Figure 4, C and E and Table S2 in File S7). This was consistent with *Mx1* accounting for ∼40% of the variance explained by the diallel, including most of the additive effects (mathematically the *Mx1* term models effects that compete with a subset of the additive and dominance diallel effects).

#### Evidence for distinct additive and nonadditive effects of Mx1 functional groups:

**Figure 5:**
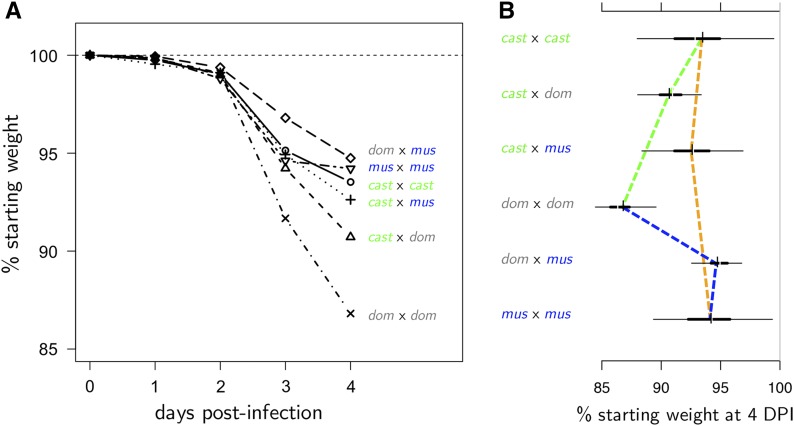
Time course of subspecies-specific *Mx1* haplotype effects on IAV-induced weight change in the diallel. (A) Predictive means of *Mx1* diplotype effects across 4 d p.i., modeled simultaneously with other diallel effects and covariates. (B) HPD intervals of *Mx1* diplotype effects on weight change on D4 p.i. Increased resistance is indicated by values further to the right. Dashed lines highlight the mode of interaction between *Mx1* haplotypes: the green line shows the additive effect of crossing *castaneus* with *domesticus*, the blue line shows the dominant effect of crossing *musculus* with *domesticus*, and the orange line shows the negligible effect of *castaneus* crossed with *musculus*.

After controlling for other diallel effects, the predicted weight loss over the course of 4 d varies in a manner consistent with *Mx1* allele combination (Figure 5A). We observed that, as expected, *domesticus* × *domesticus* crosses were predicted to have much more overall post-IAV-infection weight loss at D3 and D4 compared with all other crosses. Notably, the most protected group appeared to be the *domesticus* × *musculus* haplotype, at both D3 and D4 p.i., although the HPD intervals overlap with other *Mx1*-functional groups. The rank order of effects changes from D3 to D4 due to the dramatic slowing of weight loss in the *musculus* × *musculus* crosses from D3 to D4 compared with D2 to D3.

Although we did not observe any strain- or pairwise-specific nonadditive effects in the diallel prior to inclusion of the *Mx1* random effect, we did observe a pattern of dominance in crosses between *musculus* and *domesticus*, even as there was a pattern of additivity in the crosses between *castaneus* and *domesticus* (Figure 5B). Whereas it might be expected that host alleles from *Mx1*-null strains should act in a recessive manner, this appears not to be the case for this phenotype and time point in crosses of *castaneus* with *domesticus*, such that the functional *Mx1* allele from CAST appears to operate in an additive manner. This further supports the previous observation that the CAST *Mx1* alleles differ from the *musculus Mx1* alleles in their protective host response to IAV (Ferris *et al.* 2013).

#### Dominance and additivity of Mx1 alleles against the functional null: musculus is dominant, castaneus acts additively:

**Figure 6:**
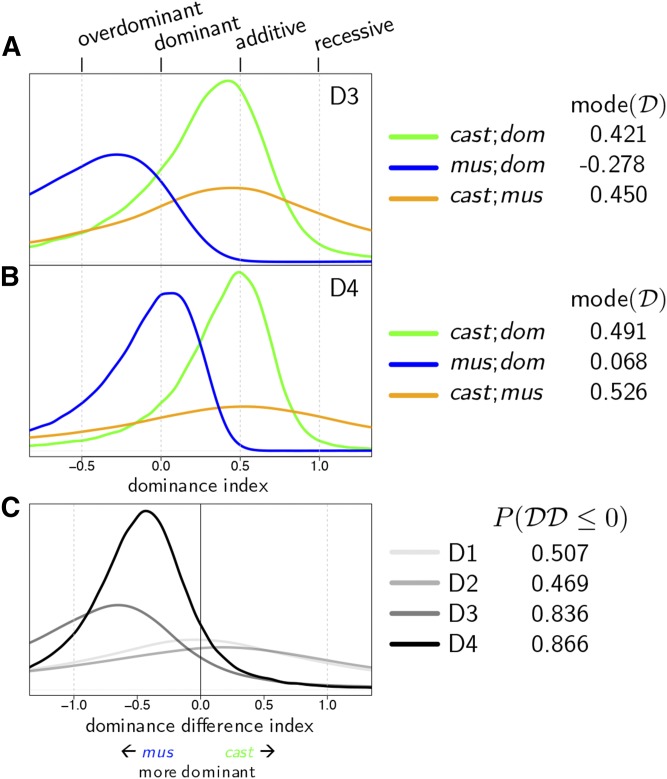
Posterior density of the dominance index on (A) D3 and (B) D4. (C) Posterior density of the dominance difference index, *i.e.*, the difference between the dominance indices of *castaneus* and *musculus*, across all 4 d.

To better characterize how the *Mx1* effects on infection response exhibit aspects of genetic dominance *vs.* genetic additivity, we estimated for each functional *Mx1* allele a dominance index, after Kacser and Burns (1981). This measures the distance between the expected phenotype of a homozygous functional allele, in our case *musculus* or *castaneus*, and the heterozygote formed with a null allele, in our case *domesticus*. On this scale, 0 denotes the functional allele being dominant to the null, 1 denotes it being recessive, and 0.5 indicates pure additivity (see *x*-axis scale in Figure 6, A and B, and more details in *Statistical Models and Methods*).

The dominance indices of the two functional *Mx1* alleles, *musculus* and *castaneus*, were sharply different (Figure 6, A and B, and Table S8 in File S7). We found that *musculus* against *domesticus* was −0.278 [= posterior mode of $D^{\left(mus;\;dom\right)};$ 80% HPD interval −2.547, 0.329] at D3 and 0.068 at D4 (−0.568, 0.380), a clear signal of *musculus* exerting classical dominance over the functional null. In contrast, the dominance index of *castaneus* against *domesticus* was 0.421 (−0.534, 0.907) and 0.491 (−0.028, 0.836) for D3 and D4, consistent with *castaneus* and the functional null being codominant (*i.e.*, having an additive relationship). The difference of the two dominance indices, whose posterior distribution is shown in Figure 6 for each time point, quantifies the distinction between *musculus* and *castaneus* more directly, putting the probability that *musculus* is more dominant than *castaneus* (*i.e.*, $P\left[D^{\left(mus;\;dom\right)}>D^{\left(cast;\;dom\right)}\right]$) at 83.6% for D3 and 86.6% for D4.

### Mx1 effects show consistent pattern in related MPPs for pre-CC and CC-RIX

**Figure 7:**
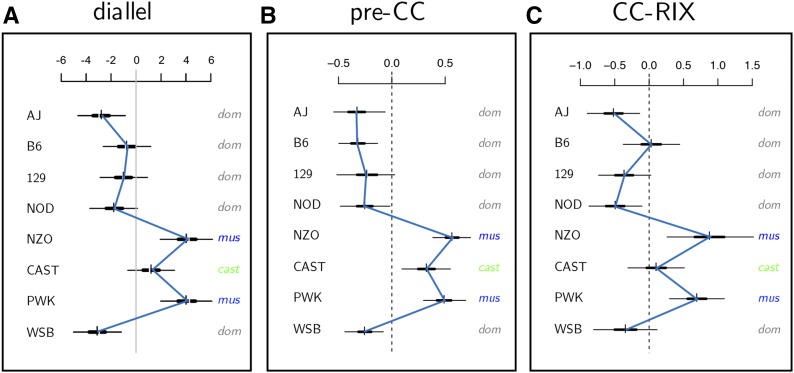
Additive CC-strain haplotype effects on IAV-induced weight loss across three CC-related populations. (A) Additive effects from the CC founder diallel of mice infected with IAV (PR8) or mock (*n*_flu_ = 393, *n*_mock_ = 131) at D4 p.i. (from Figure 1). (B) Additive strain haplotype effects at the *Mx1* locus for female pre-CC mice (*n* = 155) infected with IAV (PR8) at D4 p.i. (C) Additive strain haplotype effects at *Mx1* for female CC-RIX mice (*n* = 1402) infected with IAV (CA04) at D7 p.i. Estimates are shown as HPD intervals as described in Figure 1, with blue lines connecting posterior means. Parameter scales are given as additional IAV-induced weight loss per dose of strain in % of (A) D0, and (B and C) normalized effect size.

We examined effects associated with the *Mx1* locus in two related recombinant CC populations, the pre-CC of Ferris *et al.* (2013) and a set of CC-RIX lines first described here, and observed that the pattern of locus-specific strain haplotype effects was strikingly similar to that observed in our diallel (Figure 7). This suggests that the pattern of genome-wide additive effects in the diallel is largely driven by the effect of *Mx1* haplotypes in the founder strains. This similarity in pattern is consistent, even though the virus isolate and the peak weight loss time point differed in the CC-RIX population (CA04 human pandemic strain, D7 p.i.) compared with the diallel and pre-CC (PR8 mouse-adapted strain, D4 p.i.) (Table S4 in File S7). In all three populations, NZO and PWK alleles provide the most resistance to IAV-induced weight loss, and CAST alleles are slightly less protective. In the pre-CC, effects of AJ, B6, 129, NOD, and WSB haplotypes are all approximately the same, and clearly separated from the additive effects of strains with functional *Mx1*. In the diallel and in the CC-RIX (at *Mx1*), however, AJ and WSB haplotypes are on average more susceptible than the B6 haplotype, and there is less separation between additive effects of CAST and those from *Mx1*-null strains. The proportion of variance in weight loss explained by *Mx1* was estimated as 0.5 (95% HPD interval: 0.43, 0.54) and 0.54 (0.42, 0.63) for pre-CC and CC-RIX mice, respectively (Figures S16 and S17 in File S7). Note that an in-depth analysis of dominance indices for the *Mx1* locus was not possible in these populations because of the relatively sparse coverage of heterozygote diplotype states in the pre-CC and homozygous functional diplotype states in the CC.

## Discussion

We describe a general approach for investigating heritable effects on host susceptibility to virus-induced disease, in our case pathogenesis induced by IAV, using a diallel cross of the eight CC founder strains. The results from this diallel are informative not only in more clearly defining the genetic architecture of the host influenza response, but also prospectively: they anticipate sources of heritable variation likely to be present in the CC, the DO, and other derived experimental populations, and therefore provide a ready basis for the rational design of future studies. As an illustration of this, we demonstrate concordant effects of viral resistance locus *Mx1* across the CC founder diallel, pre-CC, and a set of CC-RIX lines.

With regard specifically to IAV pathogenesis, our study sought to better understand host genetic effects on this outcome in terms of their (1) time-dependence, (2) consistency across related populations, and (3) conditionality—for example, dependence on interactions between alleles at the same locus (dominance, at *Mx1*) or at different loci (epistasis). Regarding time-dependence (1), we found that whereas the effect of being female rather than male is evident from D1, the effects of genetics appear later, becoming evident only on D3 and then increasing through D4 p.i. Regarding consistency (2), we found that the effects of the *Mx1* alleles seen previously in the CC founders remain stable across inbred, F1, and recombinant populations. Regarding conditionality (3), we found something unexpected: evidence that the two *Mx1* functional classes, *castaneus* (CAST) and *musculus* (NZO and PWK), which were previously characterized as being functional alleles, in fact behave differently when present in the heterozygous state with susceptible *Mx1* alleles from *domesticus* (AJ, B6, 129, NOD, and WSB). Specifically, the protection conferred by the presence of a *musculus Mx1* allele is the same regardless of whether it is in the homozygote state or paired as a heterozygote with the null *domesticus* allele; the *musculus* allele is therefore dominant to *domesticus*. But for the CAST allele, when paired in the same way with *domesticus*, its protection is weakened to an extent consistent with CAST and *domesticus* being codominant, that is, having an additive relationship.

### Level of resistance to IAV among different inbred mice is conditional on IAV subtype and strain

Differences in *Mx1* function have been identified between a variety of inbred mouse strains, including the CC founders (Ferris *et al.* 2013; Xiong *et al.* 2014; Leist *et al.* 2016). Our results were largely consistent with those studies.

Notably, in their examination of the CC founders with H3N2 infection, Leist *et al.* (2016) identified AJ and WSB strains as being most susceptible, and NZO and PWK as being most resistant, which agrees with our diallel additive effects. However, in contrast with our results showing partial protection against H1N1 IAV with CAST *Mx1*, which is consistent with our prior findings in the pre-CC (Ferris *et al.* 2013); they found CAST mice, grouping with AJ and WSB, to be highly susceptible. This difference could arise for at least two reasons. First, across the influenza field, even in identical RI panels (Boon *et al.* 2009; Nedelko *et al.* 2012), host genetic effects appear to be IAV subtype specific. Second, the effectiveness of *Mx1*’s antiviral activities can vary depending on IAV subtypes (Riegger *et al.* 2015; Dittmann *et al.* 2008; Zimmermann *et al.* 2011; Mänz *et al.* 2013; Verhelst *et al.* 2012). Differentiating these two possibilities, however, is beyond the scope of this work.

Although the molecular differences in CAST *Mx1* that produce a deficient response in comparison with *musculus Mx1* have not been defined, some work has been done in inbred mice to better understand CAST (strain)-specific antiviral responses. To interpret what they saw as a unique antiviral deficiency of CAST mice, transcriptomic experiments by Leist *et al.* (2016) suggested enhanced susceptibility is due to leukocyte recruitment deficiency (relative to NZO and PWK) in the lung. In the CC founder study of Xiong *et al.* (2014), several transcriptomic differences separated the CAST response to PR8 from the that of the other strains, including differential splicing of *Irak1* and lack of *Ifng* expression at D4 p.i., which was consistent with *Ifng* deficiency observed by Earl *et al.* (2012) leading to lethal monkeypox infection of CAST mice. Because these studies were completed in inbred CAST mice, the role of CAST *Mx1* is confounded with the genome-wide differences between CAST and the other CC founders.

Thus, there are several challenges to understanding the unique IAV-resistance profile of CAST *Mx1* based on existing studies: (1) studies in inbred lines are unable to probe the overall or *Mx1*-specific dominance architecture due to a lack of heterozygosity, and (2) studies in nonrecombinant lines that identify a unique phenotype in CAST compared with other founders are unable to separate the effect of CAST *Mx1* from effects arising from the rest of the CAST genome. Our study in part circumvents these shortcomings by (1) additionally examining F1 hybrids; and (2) exploring the emerging phenotypes from an ongoing IAV-infection screen using CC-RIX, themselves F1s of RI strains.

### Complex additive effects patterns mask strong signals of dominance

In our initial analysis, we found that most of the phenotypic variation explained in infection response is driven by additive genetics with no particular signal of dominance. However, when we explicitly modeled *Mx1* status, using a term that competes with a subset of the additive and dominance diallel effects, we found that the *Mx1* functional classes act in a manner consistent with a strong dominance pattern for *musculus Mx1* (Figure 5). It seems striking that such a pattern of dominance could be underlying an apparently heavily additive effect signal.

Identifying dominance requires a good basis for comparing inbreds with hybrids. However, since the diallel is mainly composed of F1 hybrids with relatively few (8 *vs.* 54) inbreds, this basis for comparison is often weak. The BayesDiallel model handles this by considering the hybrid state as the baseline and treats the inbred state as the exception (a deviation) relevant to a minority of categories, as discussed further in Lenarcic *et al.* (2012). Inferred dominance effects are therefore vague because the data that informs them is sparse, and low estimates of dominance variance comes from absence of information rather than from information about the absence of an effect. Nonetheless, greater precision was available when considering dominance of substrain-specific *Mx1* because dominance information was pooled across multiple strains and strain pairs.

The fact that the proportion of estimated additive *vs.* nonadditive variance is influenced by model parameterization motivates careful consideration of both study design and analysis. As Huang and Mackay (2016) have recently described, model parameterizations can have critical effects on the detection of nonadditivity, with the same data strongly supporting evidence for mostly additive or mostly nonadditive effects, depending on the model. Related issues have been described at the locus level by Sabourin *et al.* (2015), who showed that when applying penalized regression to multi-SNP fine-mapping in GWAS, genotype parameterization interacts with how priors/penalties are assigned and can make biallelic dominance hard to identify in some cases. Yet even when dominance is not of interest *per se*, failure to accommodate it can disrupt estimation of additivity: in the pre-CC QTL mapping study of Phillippi *et al.* (2014), dominance signals arising from residual heterozygosity disrupted detection of an additive QTL for basal levels of CD23 (encoded by *Fcer*2); this was resolved by treating heterozygote diplotypes, whose occurrence was too sparse to be modeled, as inherently noisier via downweighting.

### Antiviral genes are expected to be dominant, but CAST Mx1 exhibits additivity

The degree of genetic dominance of host resistance factors to viral infection in humans and mice has not been thoroughly explored. In general, in the context of biochemical and immunological studies one might expect, just as with *musculus Mx1* combined with *domesticus Mx1*, that genes encoding strong-acting antivirals when combined with a null mut would be mostly dominant. In quantitative genetics, however, it is more often expected that genetic contributions will be mostly additive. In this study, at the *Mx1* locus, we observe both.

In genetic crosses of functional and null mice, major host determinants of pathogenesis are normally expected to be classified as either recessive or dominant: recessive when null results in loss of function for a host factor required for disease susceptibility, dominant when null results in loss of function for a host gene required for virus resistance. The recessive case is especially true of passive immunity gained by knockout of host genes critical to viral entry and life cycle, and has been demonstrated in a variety of studies on crop resistance (Fraser 1990; Kang *et al.* 2005; Truniger and Aranda 2009; Hashimoto *et al.* 2016) and explored in studies of the effects of CCR5 deficiency (CCR5-Δ32 deletion) in resistance to HIV infection and pathogenesis in humans (Liu *et al.* 1996; Samson *et al.* 1996; Hütter *et al.* 2009), however the degree of protection in the CCR5-Δ32 heterozygous individuals is not fully understood (Marmor *et al.* 2001; Trecarichi *et al.* 2006). The dominant case could be considered for a viral sensor, where a single inherited functional copy still provides sufficient sensitivity for viral detection and control, resembling that of an individual inheriting two copies, one from each parent. This type of dominance is best explained by the model proposed by Kacser and Burns (1981), a metabolic–enzymatic model for the architecture of dominance at specific loci, and has been explored further in studies of viral resistance in plants, such as in Fraser and Loon (1986) and Fraser (1992). The Kacser–Burns model also provides a mechanism that could in some cases give rise to additivity.

Kacser and Burns (1981) predicted that, biochemically, for most enzymes, if there is a 50% reduction in enzyme activity in the heterozygote of a null × functional cross, then in most cases the resulting phenotype will resemble that in the homozygous functional individual and the null allele would likely be characterized as operating in a “recessive” manner. According to their model, the phenotype (or “flux”) resulting from a given enzymatic pathway with multiple enzymes joined by “kinetic linking” is a summation of the change in flux due to each specific enzyme activity (“selectivity coefficient”). This means that even a dramatic change in activity for any one enzyme in a physiological system results in barely discernible changes in the system overall, as long as some functional enzyme from the locus of interest is produced.

However, the authors also describe two cases where systemic flux can be partially reduced in the heterozygote: (1) in pathways where there are exceptionally few enzymes involved in the system (this case is unlikely for an IFN-responsive antiviral pathway such as *Mx1*); and (2) in pathways where the selectivity coefficient (functional activity) of the enzyme is very low, a case termed heterozygote “indeterminacy,” which we henceforth equate to additivity. As further explored by Keightley (1996), dominance may be incomplete when less active allelic members of a series are involved in a cross with null muts, resulting in a more additive relationship; this seems most likely to explain our observation of CAST *Mx1* effects, and the lower antiviral activity of CAST *Mx1* observed in Nürnberger *et al.* (2016), discussed below, appears to support this.

### Recent work exploring CAST Mx1 antiviral deficiency

Important insights into why CAST *Mx1* might be additive come from recent functional studies. Nürnberger *et al.* (2016) engineered B6 mice expressing either the CAST-derived or A2G-derived MX1 proteins. A2G encodes an MX1 protein sequence similar to the NZO and PWK *musculus* class described in this study. CAST MX1 differs from A2G and *musculus*, with corresponding amino acid changes G83R and A222V in the G domain, which is important for enzymatic and antiviral function. Nürnberger *et al.* (2016) clearly show that CAST provides intermediate protection from IAV in their case using H7N7 (SC35M) and H5N1 (R65) viruses, and suggest that sequence changes in the CAST *Mx1* allele result in reduced enzyme stability, metabolic instability, and possibly in altered dimerization of MX1 monomers and/or changes in MX1 GTPase antiviral activity. It is unknown whether the differences they observed would lead to changes in the dominance of CAST and A2G *Mx1*, although we might expect this to be the case given our mouse infection results. We have verified that the same variants, G83R and A222V, differentiate CAST coding sequence from NZO and PWK, as in Srivastava *et al.* (2009) and using http://isvdb.unc.edu (Oreper *et al.* 2017), and that these are the only nonsynonymous variants on coding transcripts of *Mx1* that differentiate CAST from NZO and PWK. Although we see substantial protection from weight loss in CAST mice, we see a deficiency in the antiviral effects (as measured by RNA-seq viral reads in infected lungs) of CAST *Mx1* on D2 and D4 p.i. [data not shown, via RNA-seq reads from Xiong *et al.* (2014) and transcript analysis in Ferris *et al.* (2013)]. Our work motivates further functional studies of the MX1 protein using *Mx1* transgenic mice.

### Mx1-independent effects and their follow-up: new studies should leverage CAST Mx1 additivity

A substantial proportion of heritable variance in the diallel was *Mx1* independent (VarP[all|Mx1] = 33.81, Table S2 in File S7). This was broadly driven by additive genetics and both symmetric and asymmetric epistasis (*i.e.*, differing by parent-of-origin) (Figure 4, C and E). Relatedly, in our analysis of the *Mx1* locus in the CC-RIX, we estimated *Mx1*-independent effects attributable to overall genome similarity to account for 21% of phenotypic variance. Both observations suggest the presence of additional QTL that could be drawn out given a suitable follow-up design.

Consider the design of a second CC-RIX. Here our knowledge of differences in *Mx1* dominance becomes a valuable guide: prioritizing CC F1s with one copy of *musculus Mx1* would reduce power because it would cause *Mx1*-independent drivers to be masked; however, prioritizing CC F1s with one or fewer copies of *castaneus Mx1* would leave the *Mx1*-independent effects exposed and QTL underlying them more easily detected.

The inclusion of mice with a single functional *Mx1* in a mapping population provides a basis for mapping loci that modify the effect of *Mx1*, as well as mapping *Mx1*-independent loci controlling disease. Shin *et al.* (2015) showed that even the protectiveness of *Mx1* from the A2G inbred strain is conditional and depends on host genetic background. Thus, CC-RIX designs that incorporate heterozygous classes of *domesticus Mx1* crossed with either CAST *Mx1* or *musculus Mx1* can be of substantial benefit for mapping novel loci affecting infection outcomes, and at least 40% of the F1 crosses in our CC-RIX study incorporate lines which have one single copy (CAST or *musculus*) of *Mx1*.

### Practical use of the diallel in quantitative genetics

Diallels have a long history in quantitative genetics (Schmidt 1919; and references in, *e.g.*, Christie and Shattuck 1992; Verhoeven *et al.* 2006; Lenarcic *et al.* 2012). They have most commonly been used as a way to assess the relative potency of different genomes with respect to a studied trait, yielding, for example, estimates of generalized combining ability for each strain and estimates of specific combining ability for each F1. More ambitiously, they have been used to obtain an overall picture of a trait’s genetic architecture. In many respects, this picture is clearly incomplete: even within the limited genetic space spanned by the founders, the diallel shows only the effects of swapping intact haploid genomes, with no ability to see the effects of recombination. But in other respects it is comprehensive: in considering every F1 combination, one can observe evidence for types of effects—dominance, epistasis, parent-of-origin, epistasis by parent-of-origin, and all sex-specific versions thereof—that would be hard or impossible to identify in other settings, *e.g.*, outbreeding populations derived from the same set of founders.

A number of studies have sought to combine the features of a diallel with those of such derived outbred crosses to obtain a picture of genetic architecture that is in some way informed by both. These include studies that map QTL across multiple biparental (*e.g.*, F2) crosses derived from a diallel or diallel-like population (*e.g.*, Rebai and Goffinet 1993; Xu 1998; Liu and Zeng 2000; Rebaï and Goffinet 2000; Ogut *et al.* 2015) and at least one theoretical study, that of Verhoeven *et al.* (2006), examining the extent to which such information can be analyzed jointly and reconciled with data from the original diallel itself.

The goals of our study were more prospective. We use the diallel to prioritize follow-up designs in target populations that segregate genetic material from the same set of founders: the diallel provides evidence of heritable features that would be expected to exist in the CC, and that could be examined in more detail in a suitably designed CC-based experiment. Of course, a comprehensive view of IAV-resistance architecture, even within the genetic space of the CC founder genomes, would be achievable only asymptotically through countless, diverse studies; but in this, the diallel can be seen as a compass, identifying promising initial directions.

### Summary

Our study demonstrates the use of diallel crosses for identifying different types of heritable effects that can affect host responses to IAV infection. As such, we find reproducible effects of *Mx1* alleles across first order crosses and recombined populations (despite coordination between protocols being inexact), confirming our previous findings that the CAST *Mx1* allele exhibits an intermediate resistance phenotype against H1N1 strains of influenza virus (Ferris *et al.* 2013), and also identifying novel attributes of the CAST and *musculus Mx1* alleles with respect to additivity and dominance. Despite a body of literature on the effects of null mutations in *Mx1*, the importance of allelic variation at this antiviral gene is just beginning to be understood. A GWAS study published in 2011 found that *Mx1* allelic variation likely plays a role in viral disease manifestation in humans, specifically with regards to West Nile virus infection (Bigham *et al.* 2011), highlighting a need for further study of the role of natural allelic variation in *Mx1* on virus infections in future research.

## Supplementary Material

Supplemental material is available online at www.g3journal.org/lookup/suppl/doi:10.1534/g3.117.300438/-/DC1.

## Appendix A: Defining a Potential Outcomes Model of Treatment Response with Quartets

In the potential outcomes framework of Neyman (1923) and Rubin (1974), the causal effect of an applied treatment on a measured outcome in an individual *i* is defined as the difference between the outcome under treatment and the outcome that would have been observed if *i* were instead to have received the control. In our case, for some outcome measure *y*, we defined the causal effect as the infection response

$$\Delta_{i}=y_{i}^{\text{flu}}-y_{i}^{\text{mock}}\;,$$

where $y_{i}^{\text{flu}}$ and $y_{i}^{\text{mock}}$ are “potential outcomes,” one of which is observed (the factual) and other of which is unobserved (the counterfactual). Since it is impossible to observe both simultaneously, the causal effect $\Delta_{i}$ can never be measured directly (Holland 1986). It can however be estimated as

$${\hat{\Delta }}_{i}=y_{i}^{\text{flu}}-y_{i^{\prime }}^{\text{mock}}\;,$$

with the accuracy of this estimate depending on how closely $i^{\prime }$ matches *i*. Our desire for lack of bias in this measure motivates our treatment assignment being randomly assigned within a group of matched individuals.

In the treatment-response diallel, we are primarily interested not in infection response for a particular mouse but rather the expectation of this quantity for mice within a given diallel category, or more generally within a group of matched individuals *q*,

$$\Delta_{q}=\text{E}\left(y_{q}^{\text{flu}}-y_{q}^{\text{mock}}\right)=\text{E}\left(y_{q}^{\text{flu}}\right)-\text{E}\left(y_{q}^{\text{mock}}\right),$$ (A1)

where in our case *q* is defined as mice specific to a given diallel category and experimental batch. In practice it is natural to estimate this quantity as

$${\hat{\Delta }}_{q}=\frac{1}{n_{\text{flu}}}\sum_{i\in q\left[\text{flu}\right]}y_{i}^{\text{flu}}-\frac{1}{n_{\text{mock}}}\sum_{i^{\prime }\in q\left[\text{mock}\right]}y_{i^{\prime }}^{\text{mock}}\;,$$ (A2)

where q[flu] and q[mock] are, respectively, the set of mice in group *q* assigned to flu and mock treatment groups. The variance of this estimate is

$$\mathrm{Var}\left({\hat{\Delta }}_{q}\right)=\frac{\mathrm{Var}\left(y_{q}^{\text{flu}}\right)}{n_{\text{flu}}}+\frac{\mathrm{Var}\left(y_{q}^{\text{mock}}\right)}{n_{\text{mock}}},$$ (A3)

and if it is considered likely, as in this study, that the infected phenotypes will be more variable than the mock, $\mathrm{Var}\left(y^{\text{flu}}\right)>\mathrm{Var}\left(y^{\text{mock}}\right),$ then it is most efficient experimentally to devote more individuals to the infected arm than the mock arm, *i.e.*, $n_{\text{flu}}>n_{\text{mock}}.$

In our experimental design, we have flu:mock in the ratio 3:1 for each group *q*. It is therefore natural to define the unit of observation *q* as a quartet, that is, Equation A2 with $n_{\text{flu}}=3$ and $n_{\text{mock}}=1.$ This means that each diallel category can be represented by multiple quartets, corresponding to multiple observations of ${\hat{\Delta }}_{q},$ denoted in the *Statistical Models and Methods* as ${\mathrm{delta}}_{q}.$

We now note several assumptions and connections. Equation A1 equates unit-level with marginal causal effects and thereby assumes no interference between units, specifically, that mice in the same quartet do not affect each other’s outcomes; this is approximately true based on the well-established evidence that mice do not transmit H1N1 influenza virus (Lowen *et al.* 2006; Edenborough *et al.* 2012), a finding we have also verified by weight-loss profiles and RNA-seq of CC founder strains cohoused with H1N1(PR8)-infected mice (Xiong *et al.* 2014). Last, we note that the definition of ${\hat{\Delta }}_{q}$ in Equation A2 is analogous to an inverse probability-weighted causal effect estimate.

## Appendix B: Stochastic Regression Imputation of Missing Quartet Partners

The equation for the variance of ${\hat{\Delta }}_{q},$ namely Equation A3, implies that modeling the residual in Equation 5 as homoskedastic would require $n_{\text{flu}}$ and $n_{\text{mock}}$ to be constant throughout; in other words, to ensure comparable precision of infection responses, all quartets should be complete. However, in the diallel experiment, some combinations of batch and diallel category had one or more flu mice missing. In these cases, quartets were defined to have missing values that would be filled in by imputation. The imputation scheme used here corresponds to stochastic regression imputation (*e.g.*, Gelman and Hill 2006), whereby the incomplete data set is repeatedly augmented to a completed data set using sampled variates from a prediction model, each completed data set is subject to the BayesDiallel analysis described in *Statistical Models and Methods*, and then results across the completed data sets are aggregated.

Each imputation required two steps: since the target phenotype of a missing mouse, namely its p.i. weight loss, was considered potentially dependent on its D0 weight, we first imputed missing values for D0 and then imputed missing p.i. weight loss conditional on D0. In addition, at each day p.i., there was one batch/diallel category combination with one mock and four infecteds; for this case only, in each round of imputation, we created a completed quartet by randomly deleting one of the four infecteds.

The two-step stochastic regression imputation was performed as follows. Define the observed diallel data for D0 and ${\text{pct}}^{\text{day}\left[\text{flu}\right]}$ as $\text{D}0_{\text{obs}}$ and ${\text{pct}}_{\text{obs}}^{\text{day}\left[\text{flu}\right]},$ respectively, and let $\overset{\text{sim}}{∼}$ represent regression with BayesDiallel followed by stochastic regression imputation, that is, sampling from the posterior predictive. For each t=1,…,1000 round of imputation, we first impute missing D0 values as

$$\text{D}0_{\text{mis}}^{\left(t\right)}\overset{\text{sim}}{∼}\text{BayesDiallel}\left(\text{D}0_{\text{obs}}\right),$$ (B1)

where BayesDiallel is fitted as model 1 in Table 2. These imputed values are then combined with observed D0 values to give the completed set,

$$\text{D}0_{\text{complete}}^{\left(t\right)}=\left\{\text{D}0_{\text{mis}}^{\left(t\right)},\text{D}0_{\text{obs}}\right\},$$ (B2)

which are then used to impute p.i. weight loss at all time points (D1–D4) for the missing mice as

$${\text{pct}}_{\text{mis}}^{\text{day}\left[\text{flu}\right],\left(t\right)}\overset{\text{sim}}{∼}\text{BayesDiallel}\left({\text{pct}}_{\text{obs}}^{\text{day}\left[\text{flu}\right]},\text{D}0_{\text{complete}}^{\left(t\right)}\right);$$ (B3)

leading in each case to the completed flu p.i. weight loss data,

$${\text{pct}}_{\text{complete}}^{\text{day}\left[\text{flu}\right],\left(t\right)}=\left\{{\text{pct}}_{\text{mis}}^{\text{day}\left[\text{flu}\right],\left(t\right)},{\text{pct}}_{\text{obs}}^{\text{day}\left[\text{flu}\right]}\right\},$$ (B4)

and subsequent calculation of quartet-based infection response values as

$${\text{delta}}^{\text{day},\left(t\right)}=\text{Quartets}\left\{{\text{pct}}_{\text{complete}}^{\text{day}\left[\text{flu}\right],\left(t\right)},{\text{pct}}^{\text{day}\left[\text{mock}\right]}\right\}.$$ (B5)

Each of the 1000 infection response data sets is analyzed separately using BayesDiallel (model 3 in Table 2). The MCMCs from each replicate are aggregated and thinned (sampled across even intervals) and the aggregate results are reported according to the procedure outlined in Algorithm 1 in File S7.

The number of animals imputed were 33 at D1, 33 at D2, 15 at D3, and 16 at D4, in each case corresponding to a small proportion (2.8–3.1%) of the total data set. Phenotypes were not imputed for nonproductive diallel genotypes (which contain no mock or infected mice at all), *i.e.*, jk∈{NZO × CAST, NZO × PWK} (Chesler *et al.* 2008).
