# Supplementary material for: Bayesian Diallel Analysis Reveals Mx1-Dependent and Mx1-Independent Effects on Response to Influenza A Virus in Mice
Source: G3 (Bethesda). 2017 Nov 29;8(2):427–45. doi: 10.1534/g3.117.300438 (PMC5919740; doi:10.1534/g3.117.300438)
Supplement: Supplementary file 1 [file 427FileS1.pdf]

DATA UPLOAD DETAILS

Data and analysis files in this supplement include:

- File_S1_Readme.pdf - read me file providing overview of data upload.
- File_S2_Packages.tar.gz - packages used for analysis.
- File_S3_FluDiData.csv - diallel data file.
- File_S4_diallel.tar.gz - diallel analysis files.
- File_S5_preCC.tar.gz - pre-CC data and analysis files.
- File_S6_CC-RIX.tar.gz - CC-RIX data and analysis files.

After unzipping supplemental files S2, S4, S5, and S6 in a common folder, the directory structure will be as follows:

```
├── CC-RIX
...
├── diallel
├── packages
└── preCC
    └── preCCflu_happy_cache
        ├── additive
        │  └── chr16
        ├── full
        │  └── chr16
        └── genotype
            └── chr16
```

Diallel Analysis

The files that are needed to reproduce the diallel analysis are given in the "diallel" folder, which includes the raw phenotype file, `FluDiData.csv`, and the following settings files, `CategoriesToImputeD4.csv`, and `settings.config.D4`. Additional *.R, *.sh, and *.pl scripts necessary to run the analysis are included.

To run the analysis for D4 treatment response on a macOS machine with both R and Perl installed, first install prerequisites, as necessary, and then install BayesDiallel, BayesSpike, and treatmentResponseDiallel. On a bash shell, from the "diallel" directory, use the following command (example using D4):

```
bash MIMQ_Run_D4.sh &
```

When the jobs on all cores are complete, then run:

```
bash MIMQ_PostRun_D4.sh &
```

Again, when the jobs on all cores are complete, run the following to clean up temporary files:

```
bash MIMQ_cleanup.sh &
```

pre-CC Analysis

The files that are needed to reproduce the pre-CC analysis are given in the "preCC" folder. The raw phenotype file is `Flu-preCC-data.csv`, and the analysis script is `main_analysis_preCC.R`. To run the analysis, install the required prerequisites, and then INLA and Diploffect; proceed to run the analysis script from R.

CC-RIX Analysis

The files that are needed to reproduce the CC-RIX analysis are given in the "CC-RIX" folder. The raw phenotype file is `Flu-CC-RIX-data.csv`, and the analysis script is `main_analysis_CC_RIX.R`. To run the analysis, install the required prerequisites, and then INLA and Diploffect; proceed to run the analysis script from R.

Packages

The specific versions of the main analysis software packages used in this paper are provided in the "packages" folder. The packages, data and code required to reproduce the analysis in this manuscript are available as a data repository at http://dx.doi.org/10.5281/zenodo.293015, which is a static version of the GitHub repository maintained here: https://github.com/mauriziopaul/flu-diallel. The BayesDiallel and Diploffect software packages have been previously published (Lenarcic et al., 2012, *Genetics*; Zhang et al., 2014, *Genetics*), and are available at the Valdar lab website software page (http://valdarlab.unc.edu/software.html). The R package that facilitates the treatment response analysis in this paper is provided in the file `treatmentResponseDiallel_0.0.0.9000.tar.gz`, and is available at the following GitHub site: https://github.com/mauriziopaul/treatmentResponseDiallel. It can be installed according to instructions on the website. It can also be installed using the command 'R CMD INSTALL treatmentResponseDiallel_0.0.0.9000.tar.gz' in a terminal. The following prerequisite packages for treatmentResponseDiallel can be installed in a similar fashion: `configfile_0.11.tar.gz` and `cmdline_1.1.tar.gz`.

File Types

- *.R - These are R scripts used for analysis of the respective populations.
- *.pl - This is a Perl script used in the diallel analysis.
- *.sh - These are bash scripts used for the diallel analysis.
- *.RData - These files in the happy_format and happy_cache directories contain data that can be loaded in R that is necessary for the Diploffect models to run.
- *.csv - These are comma-separated files containing raw data and/or analysis settings.
- *.alleles - This file can be used to generate a full model in the happy cache.
