# Supplementary material for: Bayesian Diallel Analysis Reveals Mx1-Dependent and Mx1-Independent Effects on Response to Influenza A Virus in Mice
Source: G3 (Bethesda). 2017 Nov 29;8(2):427–45. doi: 10.1534/g3.117.300438 (PMC5919740; doi:10.1534/g3.117.300438)
Supplement: Supplementary file 7 [file 427FileS7.pdf]

## SUPPLEMENTAL FIGURES AND TABLES

**A**

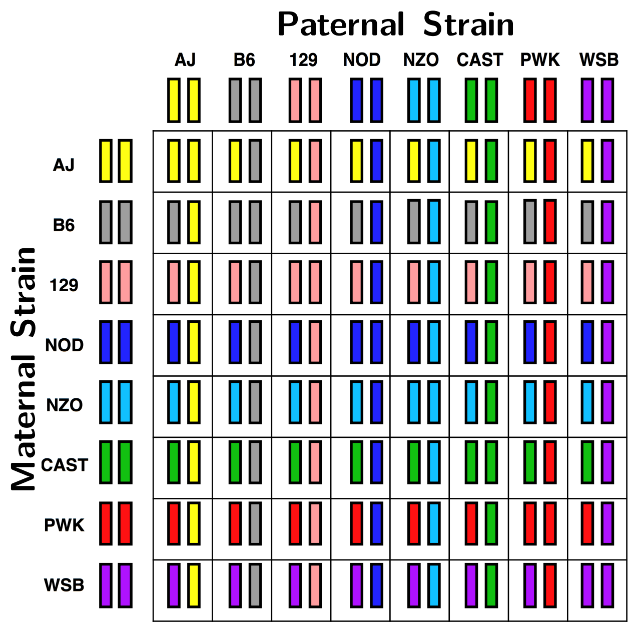


**B**

Each cell: mock / flu. Rows: dam; columns: sire.

| dam \ sire | AJ | B6 | 129 | NOD | NZO | CAST | PWK | WSB |
|---|---|---|---|---|---|---|---|---|
| AJ | 5 / 12 | 6 / 18 | 4 / 12 | 5 / 15 | 5 / 15 | 4 / 12 | 4 / 12 | 4 / 12 |
| B6 | 5 / 15 | 4 / 11 | 5 / 15 | 4 / 12 | 4 / 12 | 4 / 12 | 4 / 12 | 5 / 15 |
| 129 | 4 / 12 | 4 / 12 | 4 / 11 | 4 / 12 | 6 / 18 | 4 / 12 | 4 / 11 | 4 / 12 |
| NOD | 4 / 12 | 4 / 11 | 5 / 15 | 4 / 11 | 4 / 12 | 5 / 16 | 4 / 12 | 4 / 12 |
| NZO | 4 / 12 | 4 / 12 | 4 / 12 | 4 / 12 | 5 / 15 | - / - | - / - | 4 / 10 |
| CAST | 4 / 11 | 4 / 11 | 4 / 11 | 4 / 11 | 4 / 12 | 4 / 10 | 4 / 10 | 5 / 14 |
| PWK | 4 / 12 | 4 / 12 | 4 / 12 | 4 / 12 | 5 / 11 | 4 / 12 | 4 / 11 | 5 / 14 |
| WSB | 4 / 12 | 4 / 12 | 4 / 12 | 5 / 14 | 4 / 12 | 4 / 11 | 6 / 17 | 5 / 13 |

**Figure S1** Diallel crossing design for the influenza diallel experiment. (A) Eight inbred *Mus musculus* strains were crossed in both directions to generate animals used in this experiment. All of the animals were followed from D0 through D2. Approximately half of the animals were followed through D4, and the remaining half of the mice were sacrificed on D2 (for analysis not included in this manuscript). (B) Of the 1,043 mice in this experiment, 268 received mock treatment, and 775 received influenza (flu) inoculation, or approximately 1 mock for every 2.9 infected mice within each category. There were one hundred twenty-nine inbred mice and 914 heterozygous (hybrid F1) mice used in this study. Crosses that were not observed (marked with a "-") include NZO×CAST and NZO×PWK, for which viable offspring cannot be produced.

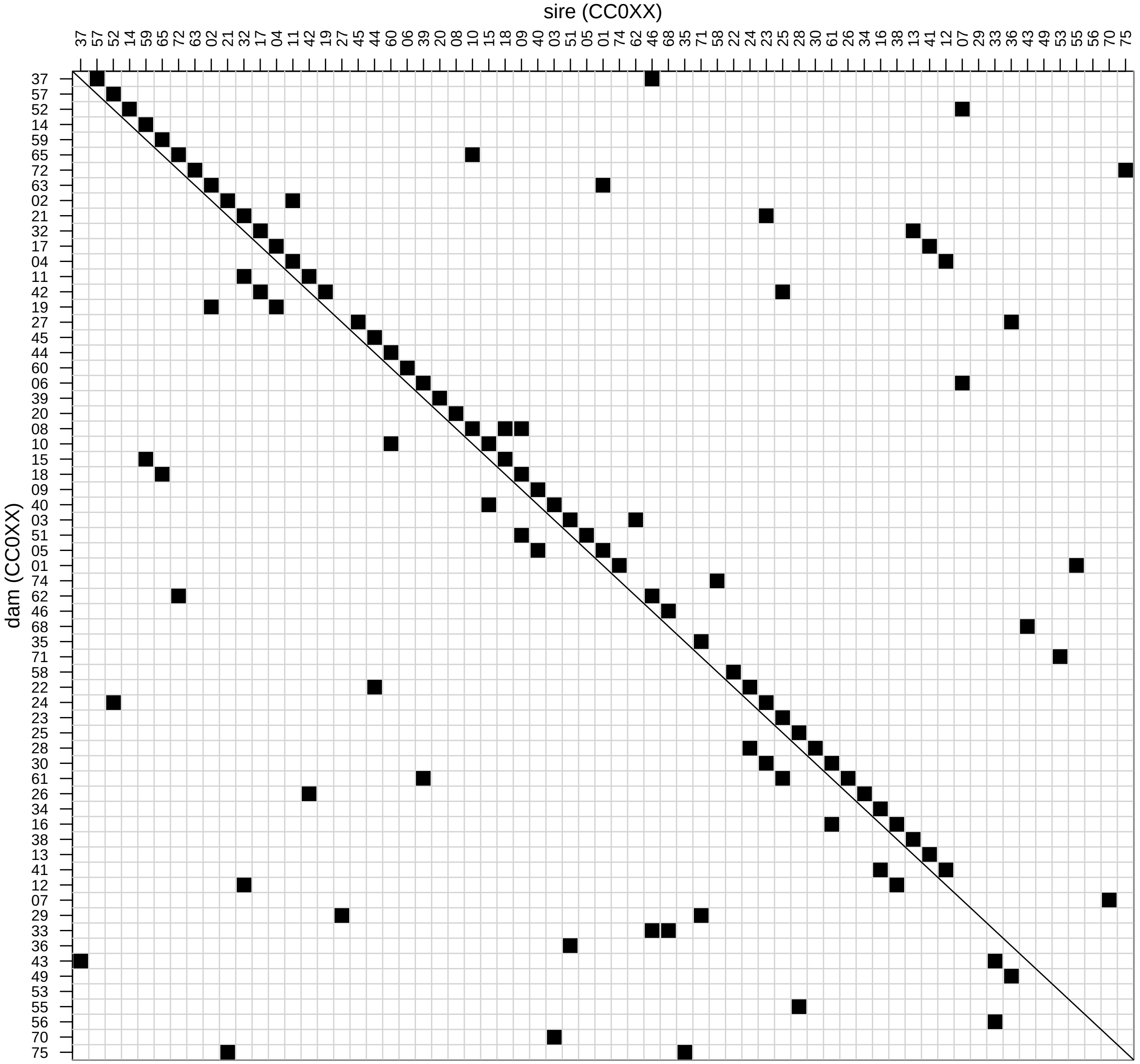


**Figure S2** CC-RIX crossing design for the influenza infection experiment. As part of a larger CC-RIX QTL mapping experiment, 65 Collaborative Cross (CC) RI strains were crossed, approximately once as dam and once as sire in a round robin breeding scheme, to generate female animals in 105 CC-RIX lines, aged 8-12 weeks, that were sacrificed on day 7 (D7) post-infection, and used in this experiment. Weights for all of the animals were measured from D0 through D7. There were 1,402 heterozygous (hybrid F1) mice used in this study.

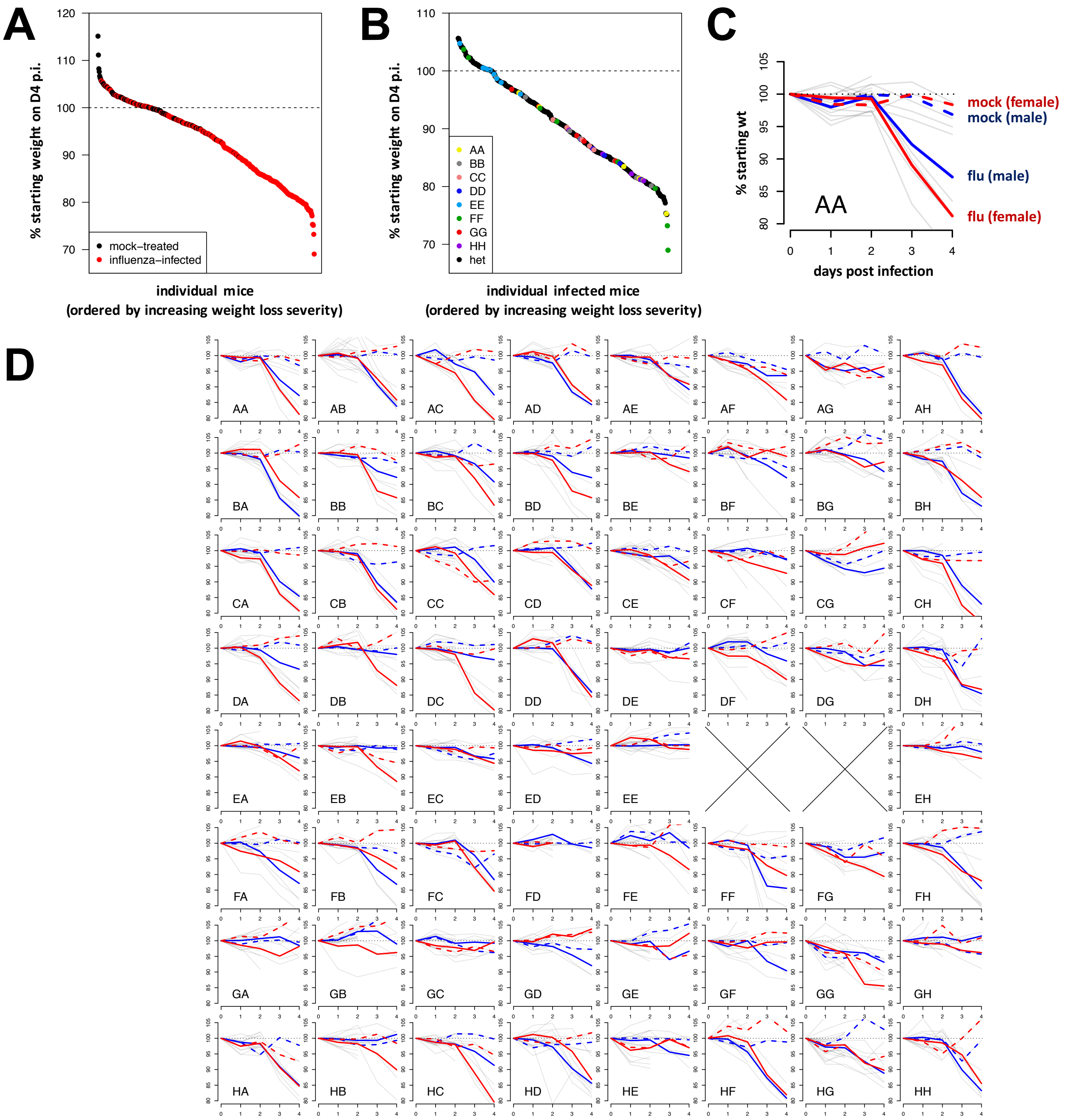


**Figure S3** The raw distribution and timecourse of mock- and flu-induced weight change in the diallel. (A) Rank-ordered distribution of individual mock- and flu-induced percent weight change of inbred and F1 animals at D4 p.i. (B) Rank-ordered distribution of inbred and F1 flu-infected percent weight change at D4 p.i. (C) An example of raw weight change curves for AA (AJ×AJ) male and female mice. (D) The panel of raw weights for the 62 possible diallel categories. In (C) and (D), y-axes indicate percent of D0 weight, and x-axes indicate day(s) post-infection, while grey lines indicate individual weight change trajectories; colored lines indicate mean male (blue) and mean female (red) weight trajectories, and among these means, colored line styles indicate mock (dashed) and influenza (solid) treatment means within each group. In (D), the large X's in EF and EG indicate non-productive F1 matings. Inbred mouse lines AJ, B6, 129, NOD, NZO, CAST, PWK, and WSB are indicated by letters A through H, respectively, with the first letter indicating maternal parentage and the second letter indicating paternal parentage.

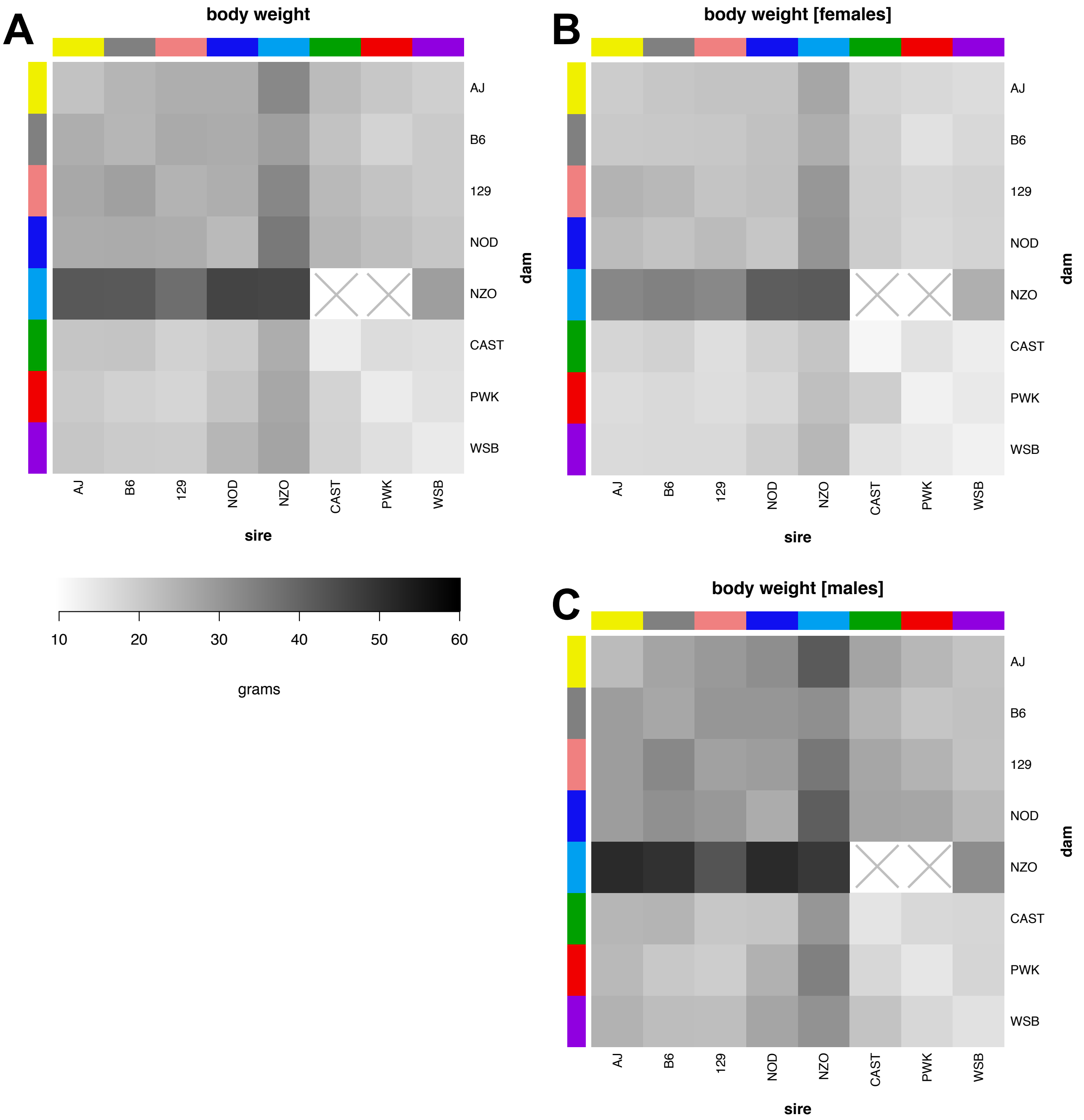


**Figure S4** Body mass in a diallel cross of inbred mice. (A) Baseline body weight (in grams) for (B) male and (C) female 8-12 week old mice (n=1,043), presented as mean values across 62 inbred and F1 diallel categories.

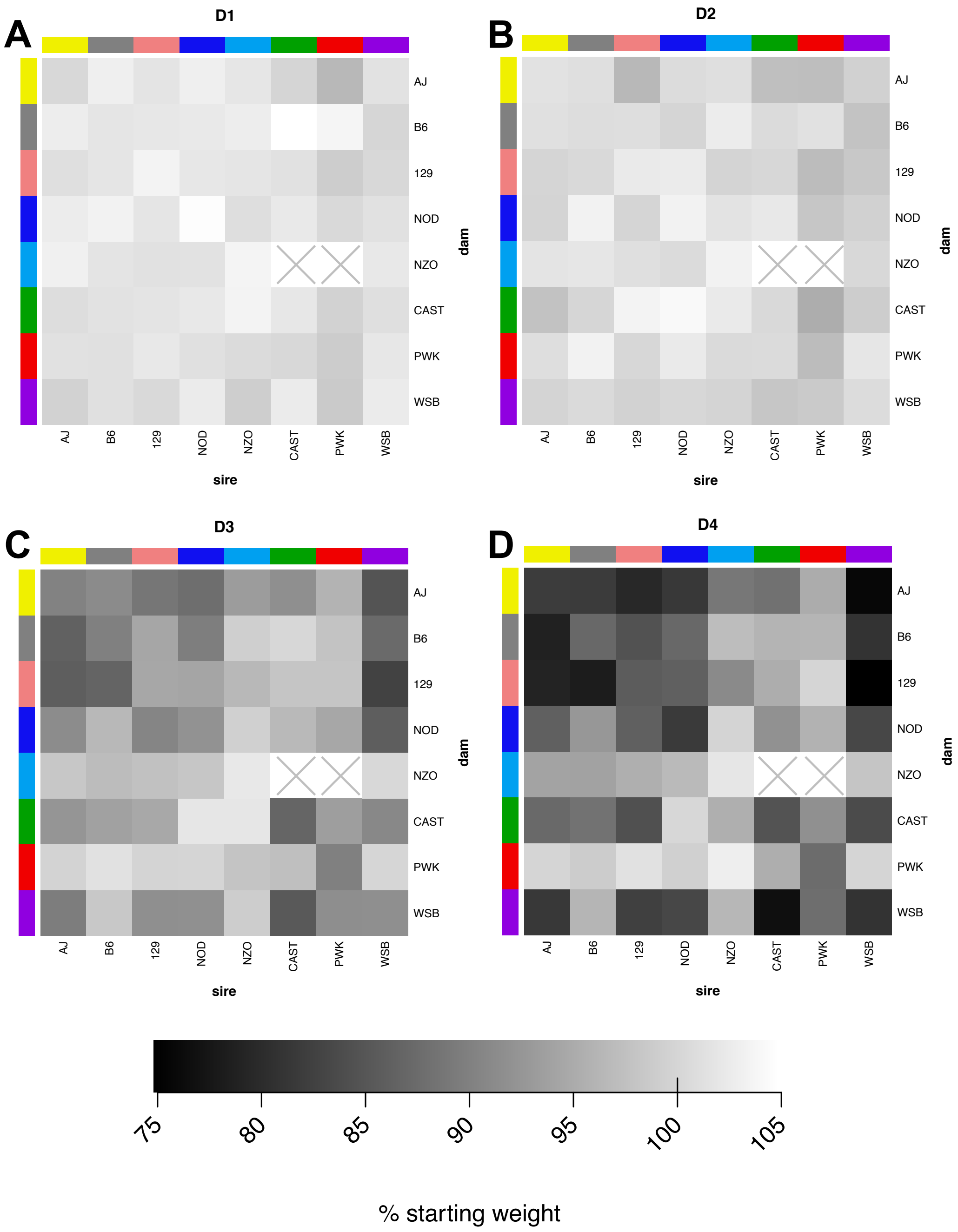


**Figure S5** Weight loss in influenza-infected mice from a diallel cross of inbred mice. (A) Change in body weight on (A) day 1 (D1), (B) D2, (C) D3, and (D) D4 post-infection, presented as mean values of percent starting weight across 62 inbred and F1 diallel categories.

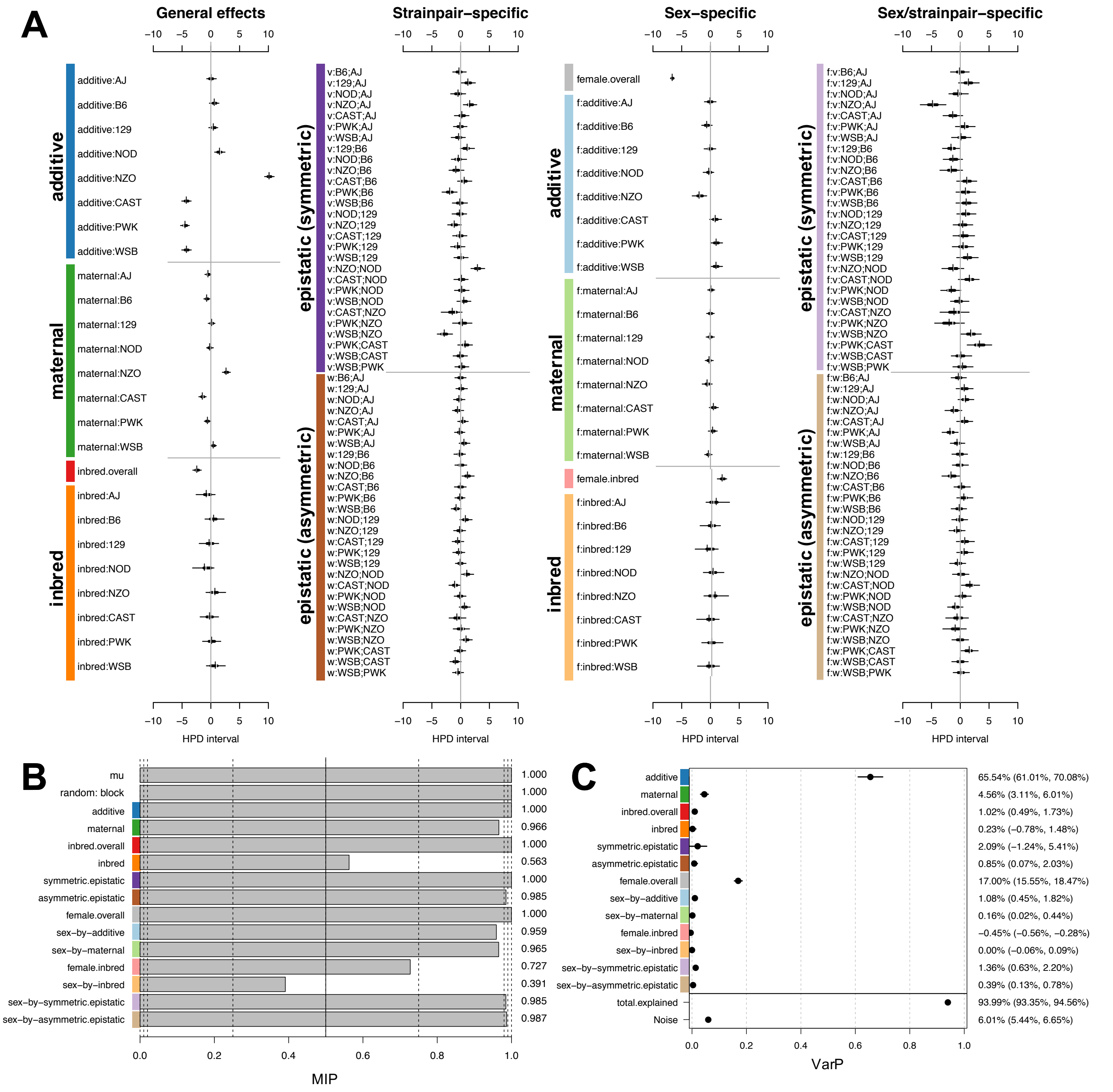


**Figure S6** Diallel effects on D0 body weight in a diallel cross of inbred mice. (A) Effect estimates for additive, maternal, inbred, and epistatic effects, including sex-specific effects, are presented as highest posterior density intervals (in grams) for adult baseline body weight in 8-12 week old mice (n=1,043). Parameters are labeled according to the methods, and intervals are presented as in Figure 4A. Symmetric epistatic, asymmetric epistatic, and sex-specific parameters are indicated by "v:", "w:", and "f:", respectively. The overall mean, $\mu$, (not shown) is 23.97 (23.56, 24.41) grams. (B) Posterior mean model inclusion probabilities (MIPs) are given for effect parameter classes. (C) Variance projection (VarPs), a generalization of heritability to the diallel effects classes, are shown for three overall effects, five random effects classes, and five corresponding sex-specific random effects classes (with posterior median and 95% HPDs).

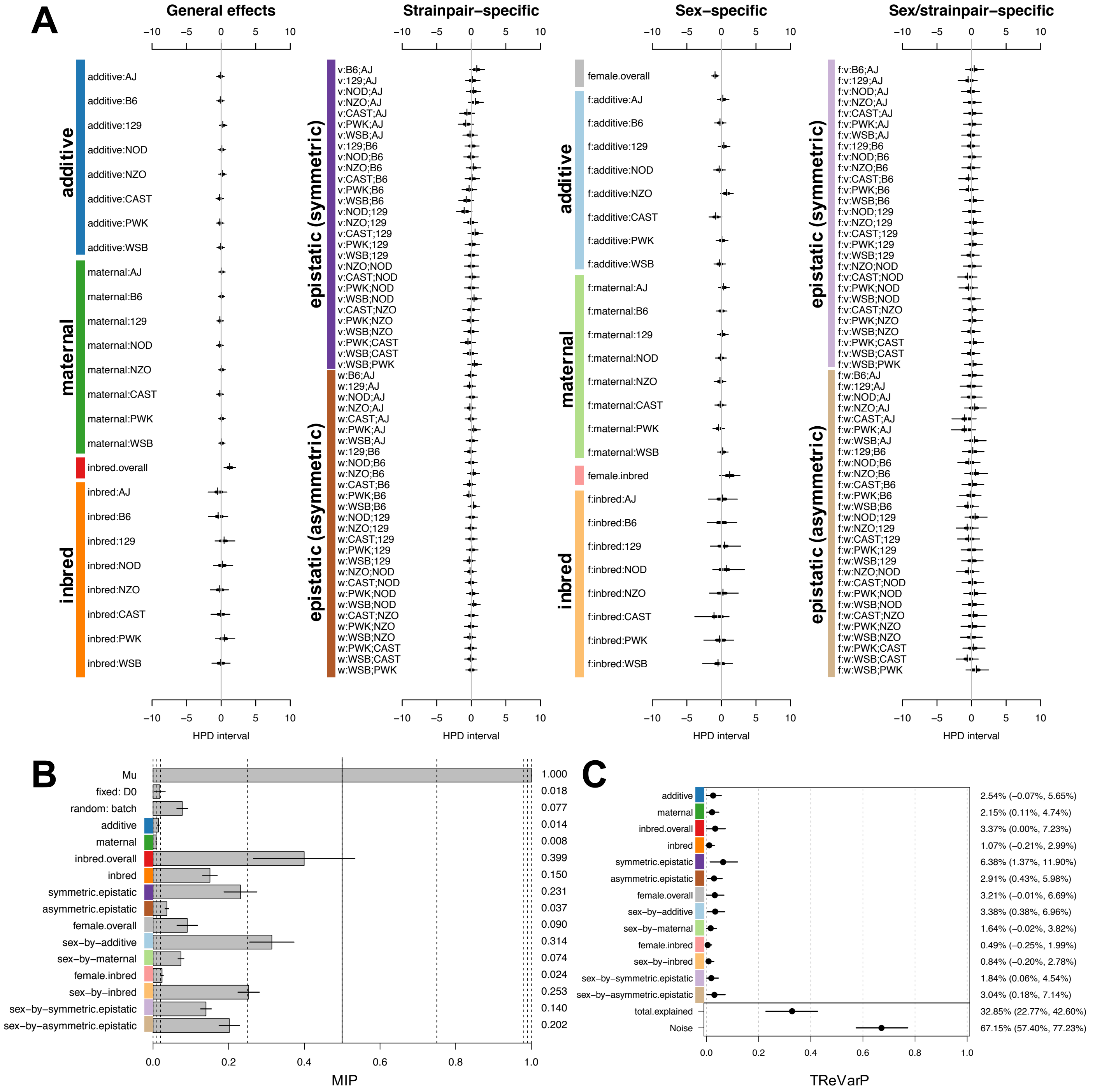


**Figure S7** Diallel effects on host infection response (weight change) at D1 post-infection, using multiple imputation matched quartets. Effect estimates for additive, maternal, inbred, and epistatic effects, including sex-specific effects, are presented as highest posterior density intervals (in percent starting weight) in 8-12 week old mice (n=1,042). Parameters are labeled according to the methods, and intervals are presented as in Figure 4A. Symmetric epistatic, asymmetric epistatic, and sex-specific parameters are indicated by "v:", "w:", and "f:", respectively. The overall treatment effect $\theta$ is -0.131% (-0.484%,0.223%). (B) Model inclusion probabilities (MIPs) are given (posterior mean $\pm$ 1 s.d.) for effect parameter classes. (C) Treatment response variance projections (TReVarPs), a generalization of heritability to the diallel effects classes, are shown for three overall effects, five random effects classes, and five corresponding sex-specific random effects classes (with posterior median and 95% HPDs).

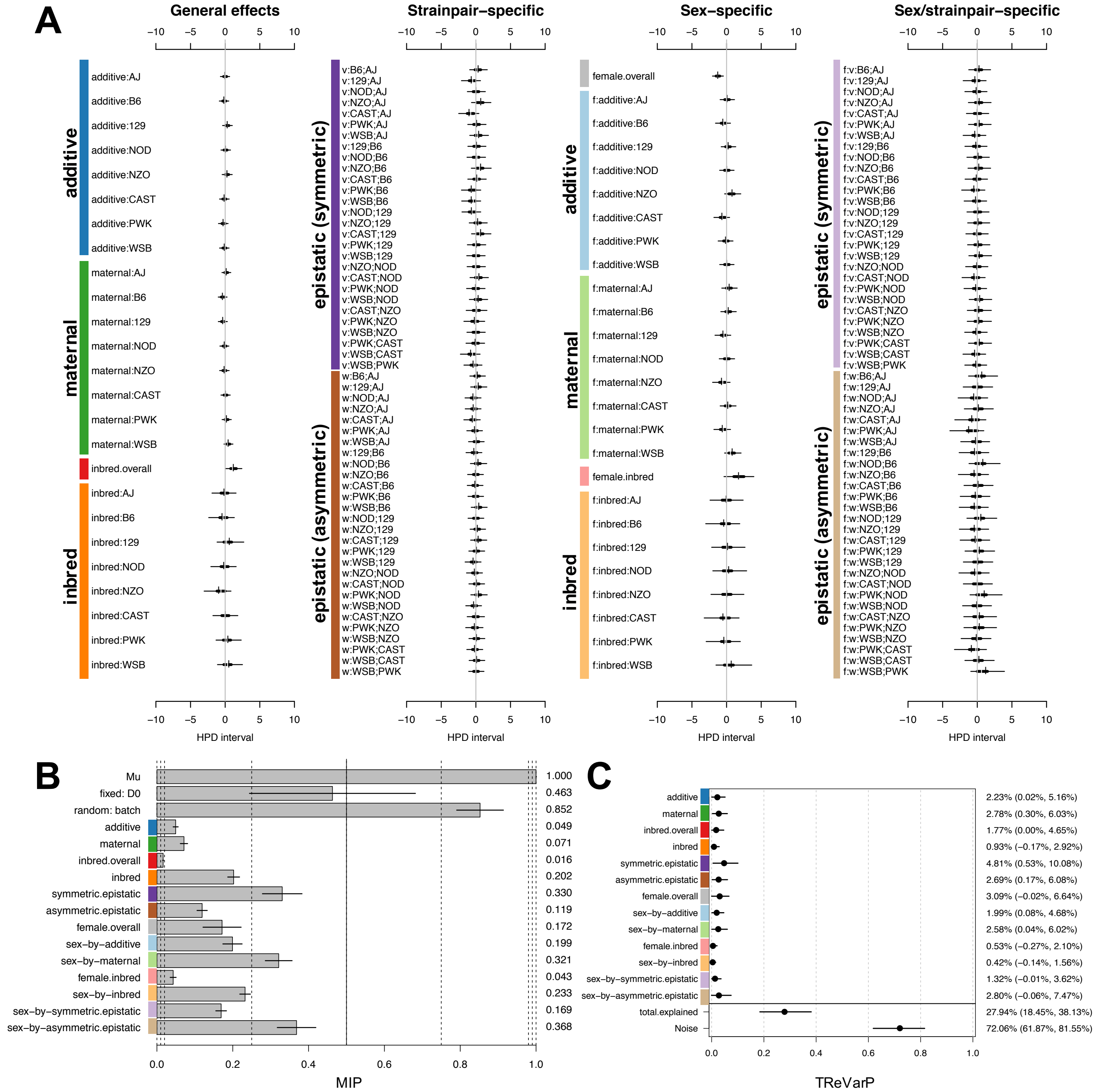


**Figure S8** Diallel effects on host infection response (weight change) at D2 post-infection, using multiple imputation matched quartets. Effect estimates for additive, maternal, inbred, and epistatic effects, including sex-specific effects, are presented as highest posterior density intervals (in percent starting weight) in 8-12 week old mice (n=1,042). Parameters are labeled according to the methods, and intervals are presented as in Figure 4A. Symmetric epistatic, asymmetric epistatic, and sex-specific parameters are indicated by "v:", "w:", and "f:", respectively. The overall treatment effect $\theta$ is -0.833% (-1.328%,-0.318%). (B) Model inclusion probabilities (MIPs) are given (posterior mean $\pm$ 1 s.d.) for effect parameter classes. (C) Treatment response variance projections (TReVarPs), a generalization of heritability to the diallel effects classes, are shown for three overall effects, five random effects classes, and five corresponding sex-specific random effects classes (with posterior median and 95% HPDs).

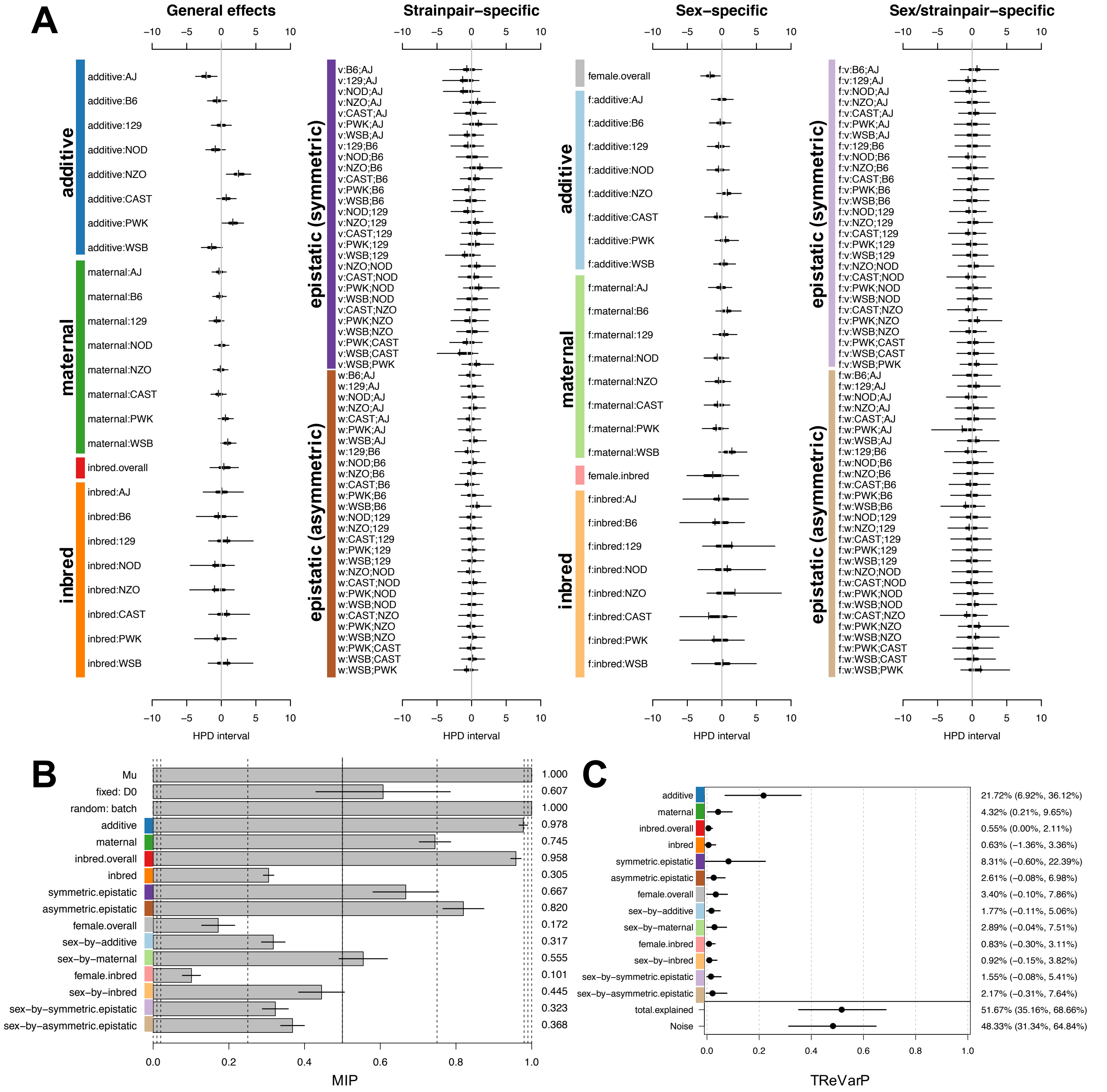


**Figure S9** Diallel effects on host infection response (weight change) at D3 post-infection, using multiple imputation matched quartets. Effect estimates for additive, maternal, inbred, and epistatic effects, including sex-specific effects, are presented as highest posterior density intervals (in percent starting weight) in 8-12 week old mice (n=514). Parameters are labeled according to the methods, and intervals are presented as in Figure 4A. Symmetric epistatic, asymmetric epistatic, and sex-specific parameters are indicated by "v:", "w:", and "f:", respectively. The overall treatment effect $\theta$ is -5.594% (-6.470%, -4.729%). (B) Model inclusion probabilities (MIPs) are given (posterior mean $\pm$ 1 s.d.) for effect parameter classes. (C) Treatment response variance projections (TReVarPs), a generalization of heritability to the diallel effects classes, are shown for three overall effects, five random effects classes, and five corresponding sex-specific random effects classes (with posterior median and 95% HPDs).

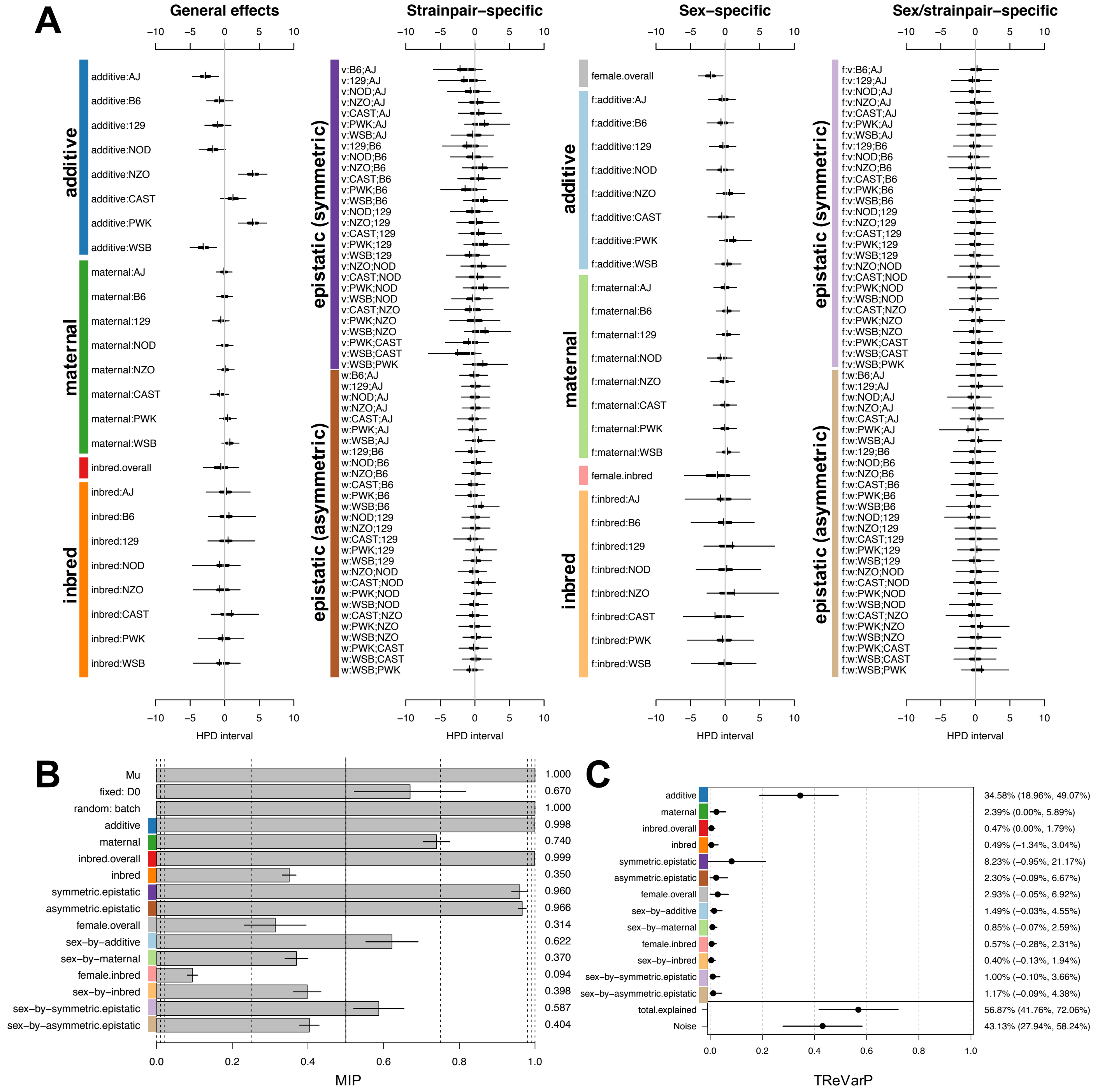


**Figure S10** Diallel effects on host infection response (weight change) at D4 post-infection, using multiple imputation matched quartets. Effect estimates for additive, maternal, inbred, and epistatic effects, including sex-specific effects, are presented as highest posterior density intervals (in percent starting weight) in 8-12 week old mice (n=513). Parameters are labeled according to the methods, and intervals are presented as in Figure 4A. Symmetric epistatic, asymmetric epistatic, and sex-specific parameters are indicated by "v:", "w:", and "f:", respectively. The overall treatment effect $\theta$ is -8.849% (-9.920%, -7.779%). (B) Model inclusion probabilities (MIPs) are given (posterior mean $\pm$ 1 s.d.) for effect parameter classes. (C) Treatment response variance projections (TReVarPs), a generalization of heritability to the diallel effects classes, are shown for three overall effects, five random effects classes, and five corresponding sex-specific random effects classes (with posterior median and 95% HPDs).

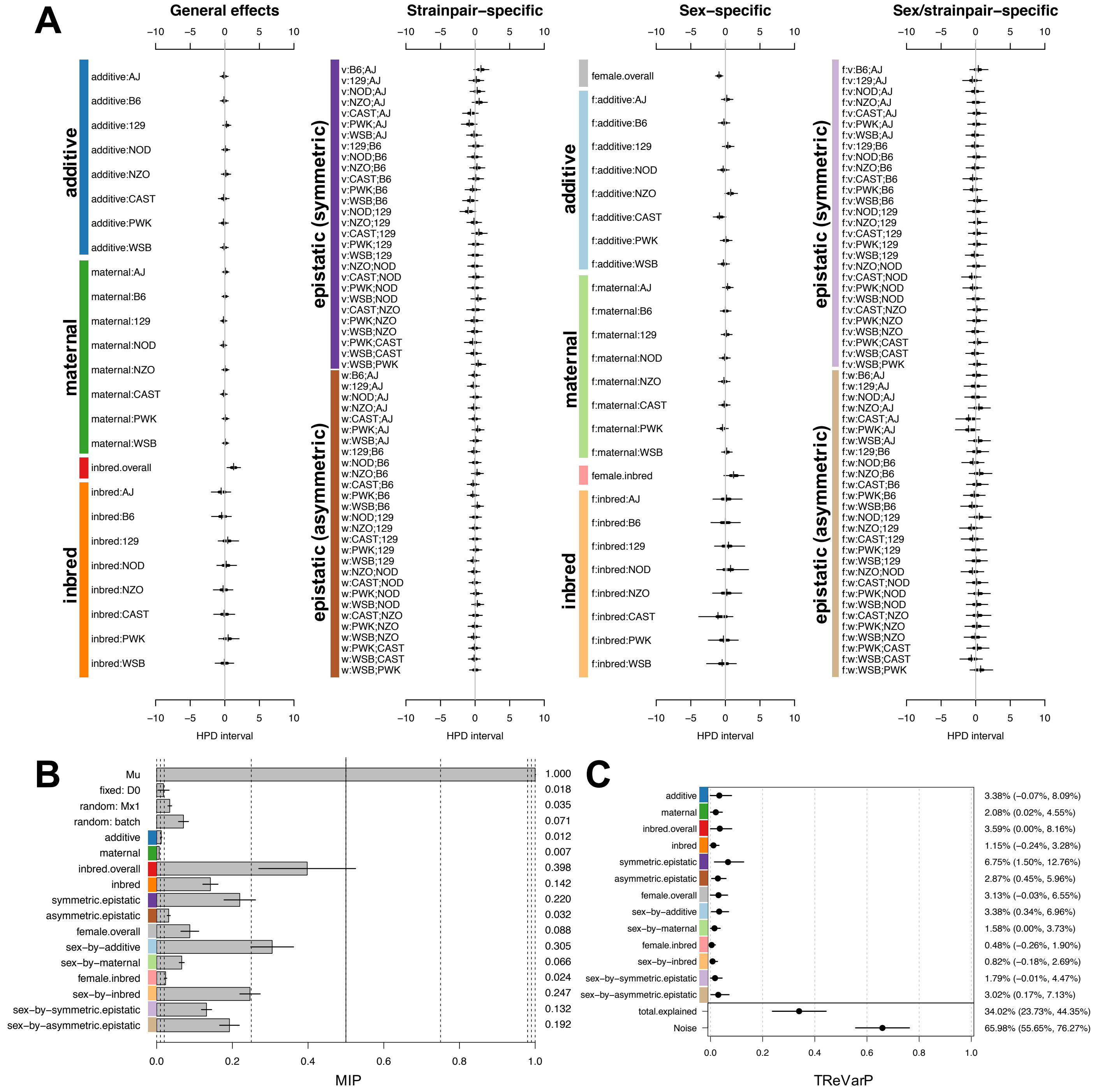


**Figure S11** Diallel effects on host infection response (weight change) at D1 post-infection, using multiple imputation matched quartets and accounting for *Mx1*. Effect estimates for additive, maternal, inbred, and epistatic effects, including sex-specific effects, are presented as highest posterior density intervals (in percent starting weight) in 8-12 week old mice (n=1,042) after including random effect $u^{(Mx1 \text{ diplo})}$. Parameters are labeled according to the methods, and intervals are presented as in Figure 4A. Symmetric epistatic, asymmetric epistatic, and sex-specific parameters are indicated by "v:", "w:", and "f:", respectively. The overall treatment effect $\theta$ is -0.193% (-1.128%, 0.707%). (B) Model inclusion probabilities (MIPs) are given (posterior mean $\pm$ 1 s.d.) for effect parameter classes. (C) Treatment response variance projections (TReVarPs), a generalization of heritability to the diallel effects classes, are shown for three overall effects, five random effects classes, and five corresponding sex-specific random effects classes (with posterior median and 95% HPDs).

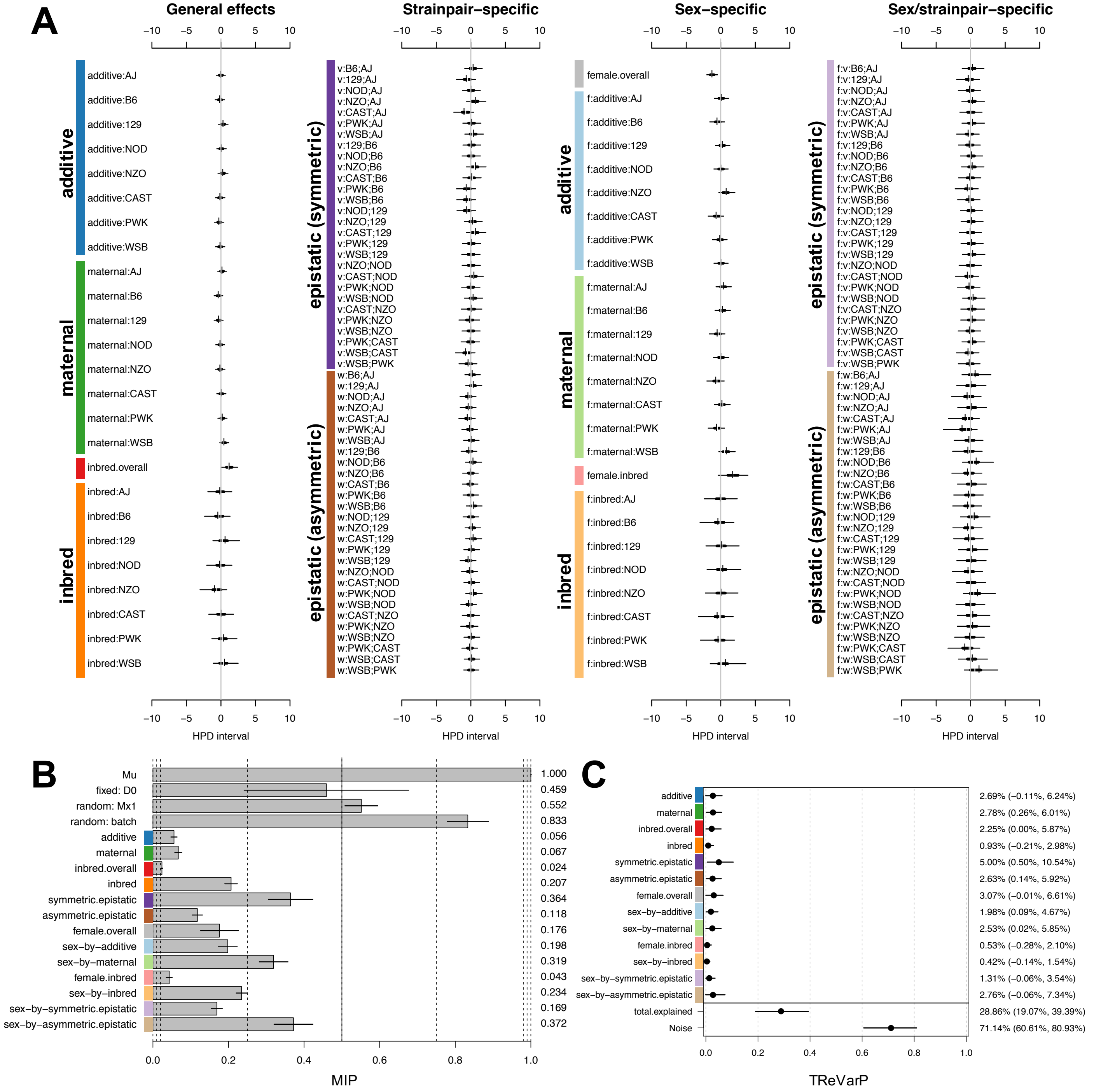


**Figure S12** Diallel effects on host infection response (weight change) at D2 post-infection, using multiple imputation matched quartets and accounting for *Mx1*. Effect estimates for additive, maternal, inbred, and epistatic effects, including sex-specific effects, are presented as highest posterior density intervals (in percent starting weight) in 8-12 week old mice (n=1,042) after including random effect $u^{(Mx1\ \text{diplo})}$. Parameters are labeled according to the methods, and intervals are presented as in Figure 4A. Symmetric epistatic, asymmetric epistatic, and sex-specific parameters are indicated by "v:", "w:", and "f:", respectively. The overall treatment effect $\theta$ is -0.925% (-1.991%, 0.113%). (B) Model inclusion probabilities (MIPs) are given (posterior mean $\pm$ 1 s.d.) for effect parameter classes. (C) Treatment response variance projections (TReVarPs), a generalization of heritability to the diallel effects classes, are shown for three overall effects, five random effects classes, and five corresponding sex-specific random effects classes (with posterior median and 95% HPDs).


G3 Genes | Genomes | Genetics

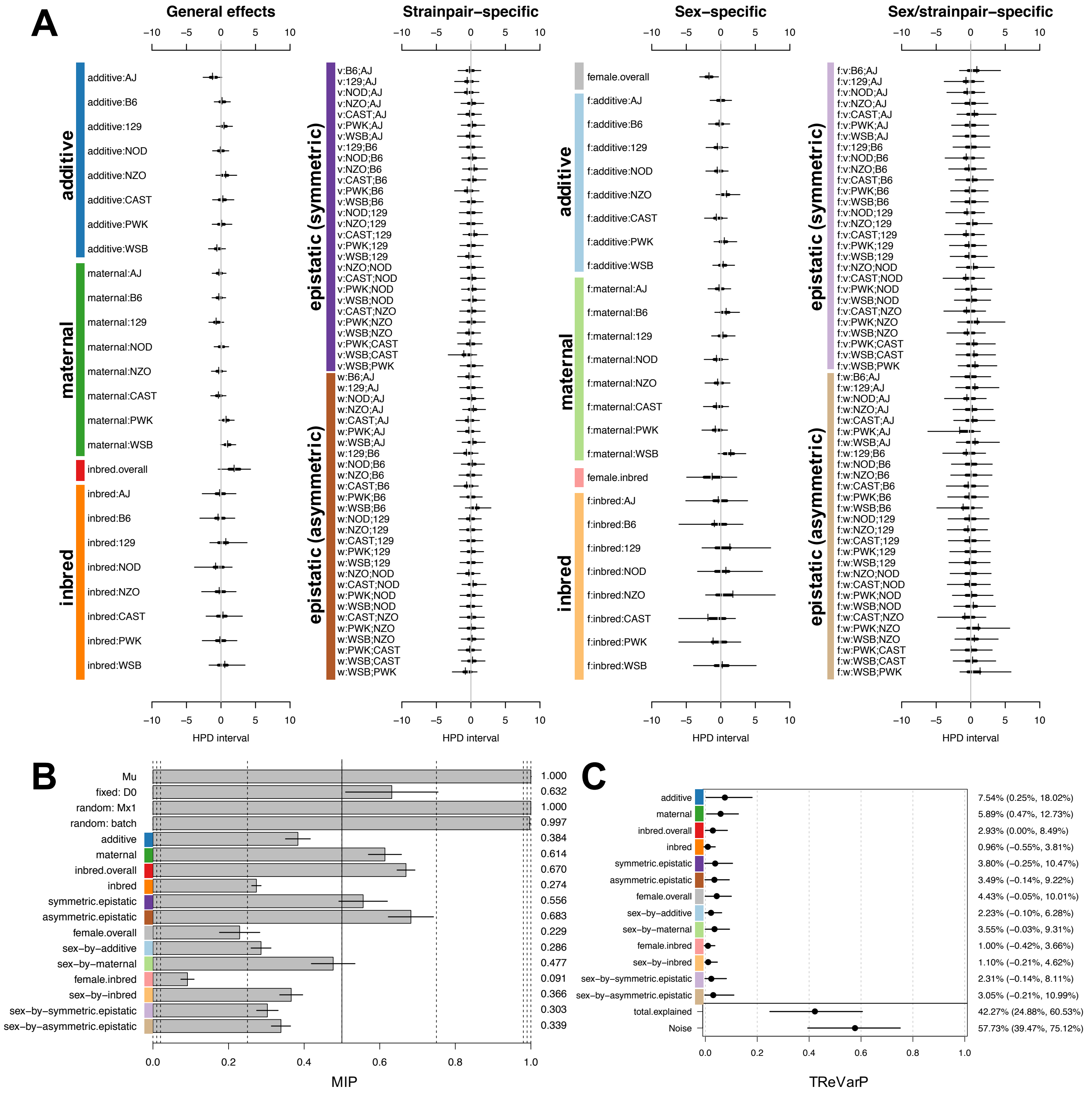


**Figure S13** Diallel effects on host infection response (weight change) at D3 post-infection, using multiple imputation matched quartets and accounting for *Mx1*. Effect estimates for additive, maternal, inbred, and epistatic effects, including sex-specific effects, are presented as highest posterior density intervals (in percent starting weight) in 8-12 week old mice (n=514) after including random effect $u^{(Mx1\ \text{diplo})}$. Parameters are labeled according to the methods, and intervals are presented as in Figure 4A. Symmetric epistatic, asymmetric epistatic, and sex-specific parameters are indicated by "v:", "w:", and "f:", respectively. The overall treatment effect $\theta$ is -5.429% (-7.675%, -3.102%). (B) Model inclusion probabilities (MIPs) are given (posterior mean $\pm$ 1 s.d.) for effect parameter classes. (C) Treatment response variance projections (TReVarPs), a generalization of heritability to the diallel effects classes, are shown for three overall effects, five random effects classes, and five corresponding sex-specific random effects classes (with posterior median and 95% HPDs).

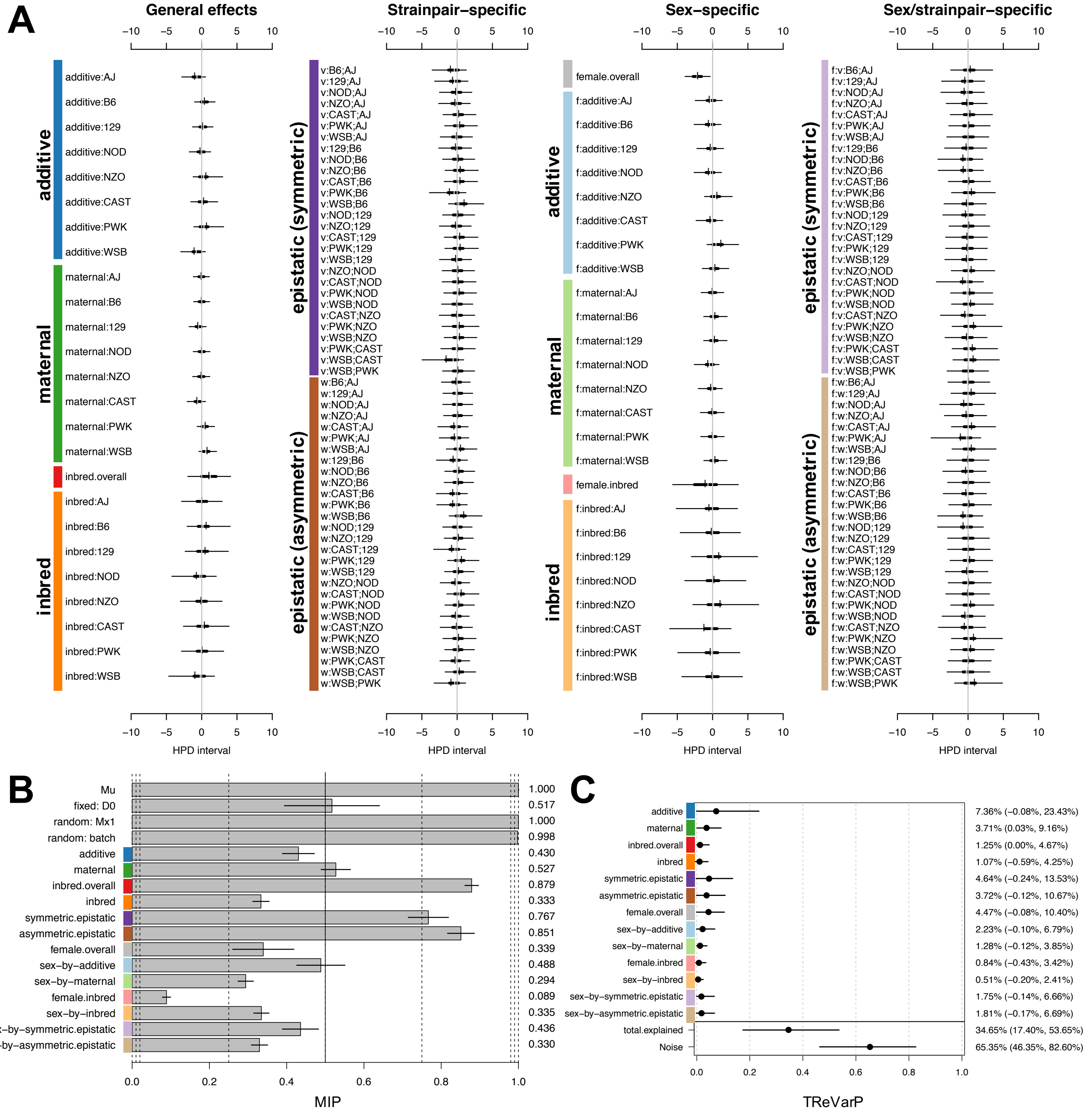


**Figure S14** Diallel effects on host infection response (weight change) at D4 post-infection, using multiple imputation matched quartets and accounting for *Mx1*. Effect estimates for additive, maternal, inbred, and epistatic effects, including sex-specific effects, are presented as highest posterior density intervals (in percent starting weight) in 8-12 week old mice (n=513) after including random effect $u^{(Mx1\ \mathrm{diplo})}$. Parameters are labeled according to the methods, and intervals are presented as in Figure 4A. Symmetric epistatic, asymmetric epistatic, and sex-specific parameters are indicated by "v:", "w:", and "f:", respectively. The overall treatment effect $\theta$ is -7.986% (-11.202%, -4.147%). (B) Model inclusion probabilities (MIPs) are given (posterior mean $\pm$ 1 s.d.) for effect parameter classes. (C) Treatment response variance projections (TReVarPs), a generalization of heritability to the diallel effects classes, are shown for three overall effects, five random effects classes, and five corresponding sex-specific random effects classes (with posterior median and 95% HPDs).

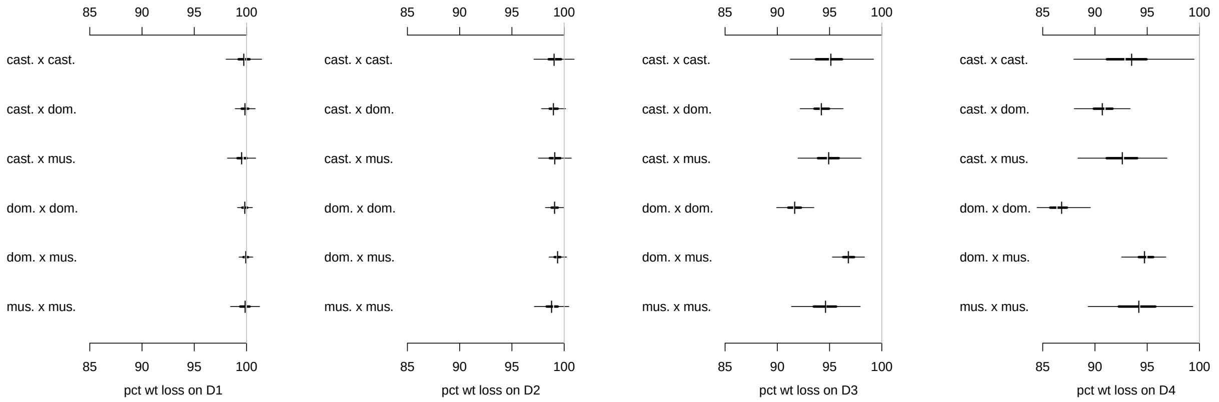


**Figure S15** Timecourse of posterior predictive estimates of for *Mx1* diplotype mean effects across four days post-infection. From left to right, predictive estimates of IAV-induced weight change are provided for D1, D2, D3 and D4 p.i.

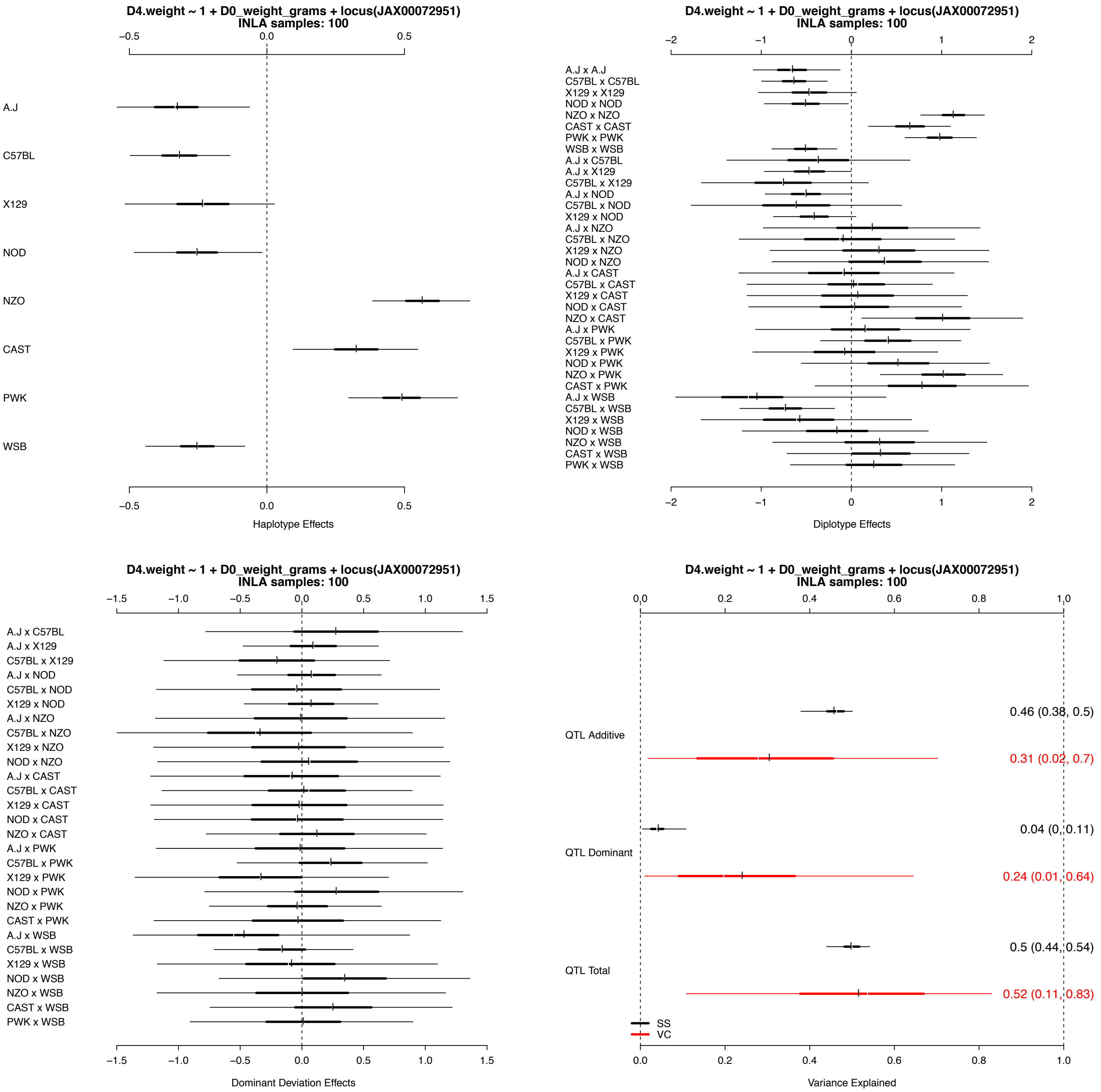


**Figure S16** *Mx1* effects and proportion of variance explained on weight loss in the pre-CC. [Top Left] Additive *Mx1* haplotype effects in pre-CC mice (n=155) infected with IAV (PR8) at day 4 post-infection. [Top Right] Bayesian credible intervals of the posterior predictive distribution of mice with *Mx1* diplotype. [Bottom Left] Dominance deviation *Mx1* effects in the pre-CC mice. Wide intervals are indicative of the low levels of heterozygosity observed in the mostly inbred pre-CC mice. [Bottom Right] Bayesian credible intervals of the posterior predictive distribution of the proportion of the phenotypic variance explained by the *Mx1* additive effects, dominant effects and the combination of both effects. Black intervals (VC) are based on the posterior samples of the variance components. Red intervals (SS) are based on the posterior samples of sums of squares estimators. SS estimators are (1) not dependent on variance component estimates, which tend to be disperse, and (2) are based on the observed strains and crosses, rather than all possible strains/crosses, with (1) and (2) resulting in much narrower confidence intervals.

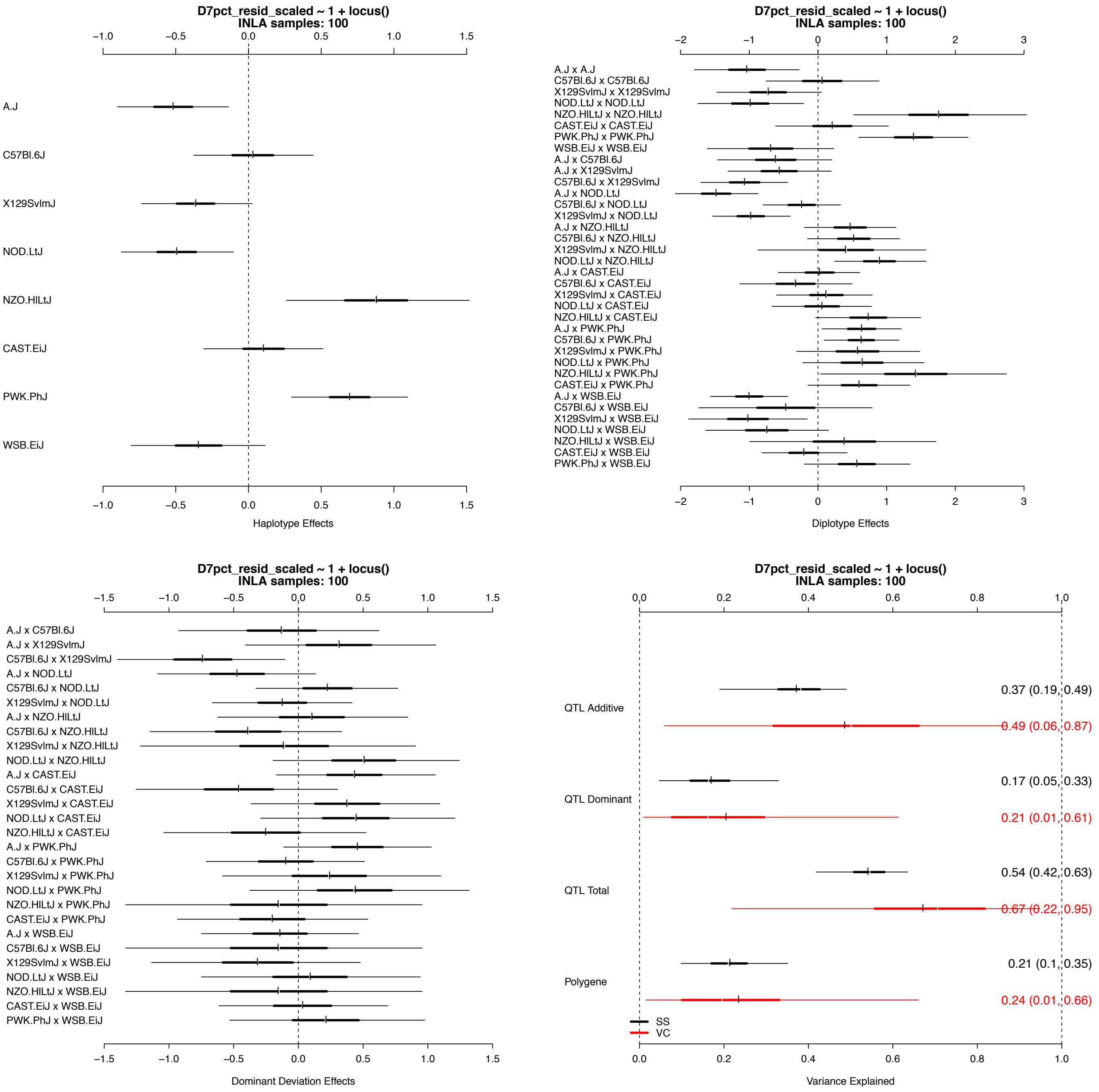


**Figure S17** *Mx1* allele effects and proportion of variance explained on weight loss in the CC-RIX. [Top Left] Additive *Mx1* haplotype effects in CC-RIX mice (n=1,402) infected with IAV (CA04) at day 7 post-infection. [Top Right] Bayesian credible intervals of the posterior predictive distribution of mice with *Mx1* diplotype. [Bottom Left] Dominance deviation *Mx1* effects in the CC-RIX mice. Narrower intervals compared to observed in pre-CC is indicative of the high levels of heterozygosity CC-RIX mice. [Bottom Right] Bayesian credible intervals of the posterior predictive distribution of the proportion of the phenotypic variance explained by the *Mx1* additive effects, dominant effects, the combination of both *Mx1* effects, and cumulative effects of other loci captured in the relationship matrix. In the CC-RIX, the relationship matrix models the expected increased phenotypic correlation between CC-RIX mice that share a single CC parent. Black intervals (VC) are based on the posterior samples of the variance components. Red intervals (SS) are based on the posterior samples of sums of squares estimators. SS estimators are (1) not dependent on variance component estimates, which tend to be disperse, and (2) are based on the observed strains and crosses, rather than all possible strains/crosses, with (1) and (2) resulting in much narrower confidence intervals.

**Table S1 Proportion of variance in IAV-induced weight loss attributable to diallel effect classes across 4 days post-infection.** Part C of **Figure S7** through **Figure S10** show Bayesian confidence interval plots of treatment response variance projection (TReVarP) values presented here.

| Effect Class [a] | D1 | 0.025 | 0.975 | D2 | 0.025 | 0.975 | D3 | 0.025 | 0.975 | D4 | 0.025 | 0.975 |
|---|---|---|---|---|---|---|---|---|---|---|---|---|
| additive | 0.0229 | -0.0007 | 0.0565 | 0.0197 | 0.0002 | 0.0516 | 0.2163 | 0.0692 | 0.3612 | 0.3477 | 0.1896 | 0.4907 |
| maternal | 0.0192 | 0.0011 | 0.0474 | 0.0253 | 0.0030 | 0.0603 | 0.0381 | 0.0021 | 0.0965 | 0.0196 | 0.0000 | 0.0589 |
| inbred (overall) | 0.0306 | 0.0000 | 0.0723 | 0.0142 | 0.0000 | 0.0465 | 0.0025 | 0.0000 | 0.0211 | 0.0021 | 0.0000 | 0.0179 |
| inbred | 0.0082 | -0.0021 | 0.0299 | 0.0064 | -0.0017 | 0.0292 | 0.0031 | -0.0136 | 0.0336 | 0.0023 | -0.0134 | 0.0304 |
| symmetric epistatic | 0.0605 | 0.0137 | 0.1190 | 0.0433 | 0.0053 | 0.1008 | 0.0655 | -0.0060 | 0.2239 | 0.0692 | -0.0095 | 0.2117 |
| asymmetric epistatic | 0.0264 | 0.0043 | 0.0598 | 0.0233 | 0.0017 | 0.0608 | 0.0203 | -0.0008 | 0.0698 | 0.0163 | -0.0009 | 0.0667 |
| female (overall) | 0.0296 | -0.0001 | 0.0669 | 0.0282 | -0.0002 | 0.0664 | 0.0299 | -0.0010 | 0.0786 | 0.0256 | -0.0005 | 0.0692 |
| sex-by-additive | 0.0309 | 0.0038 | 0.0696 | 0.0169 | 0.0008 | 0.0468 | 0.0131 | -0.0011 | 0.0506 | 0.0103 | -0.0003 | 0.0455 |
| sex-by-maternal | 0.0143 | -0.0002 | 0.0382 | 0.0224 | 0.0004 | 0.0602 | 0.0230 | -0.0004 | 0.0751 | 0.0059 | -0.0007 | 0.0259 |
| female inbred (overall) | 0.0024 | -0.0025 | 0.0199 | 0.0027 | -0.0027 | 0.0210 | 0.0047 | -0.0030 | 0.0311 | 0.0027 | -0.0028 | 0.0231 |
| sex-by-inbred | 0.0054 | -0.0020 | 0.0278 | 0.0024 | -0.0014 | 0.0156 | 0.0034 | -0.0015 | 0.0382 | 0.0012 | -0.0013 | 0.0194 |
| sex-by-symm. epis. | 0.0152 | 0.0006 | 0.0454 | 0.0100 | -0.0001 | 0.0362 | 0.0089 | -0.0008 | 0.0541 | 0.0052 | -0.0010 | 0.0366 |
| sex-by-asymm. epis. | 0.0259 | 0.0018 | 0.0714 | 0.0218 | -0.0006 | 0.0747 | 0.0119 | -0.0031 | 0.0764 | 0.0059 | -0.0009 | 0.0438 |
| total explained | 0.3276 | 0.2277 | 0.4260 | 0.2775 | 0.1845 | 0.3813 | 0.5164 | 0.3516 | 0.6866 | 0.5705 | 0.4176 | 0.7206 |
| noise | 0.6724 | 0.5740 | 0.7723 | 0.7225 | 0.6187 | 0.8155 | 0.4836 | 0.3134 | 0.6484 | 0.4295 | 0.2794 | 0.5824 |

[a] To indicate the relative contribution of different diallel effects on host response to IAV, TreVarPs are given for overall effects (female, inbred, female inbred) and strain/strainpair-specific effects (additive, maternal, inbred, symmetric epistatic, asymmetric epistatic) on D1delta through D4delta (median and 95% HPD intervals).

**Table S2 Proportion of variance in IAV-induced weight loss attributable to diallel effect classes across 4 days post-infection, after accounting for *Mx1* diplotype.** Part C of **Figure S11** through **Figure S14** show Bayesian confidence interval plots of treatment response variance projection (TReVarP) values presented here.

| Effect Class [a] | D1 | 0.025 | 0.975 | D2 | 0.025 | 0.975 | D3 | 0.025 | 0.975 | D4 | 0.025 | 0.975 |
|---|---|---|---|---|---|---|---|---|---|---|---|---|
| additive | 0.0292 | -0.0007 | 0.0809 | 0.0233 | -0.0011 | 0.0624 | 0.0631 | 0.0025 | 0.1802 | 0.0496 | -0.0008 | 0.2343 |
| maternal | 0.0188 | 0.0002 | 0.0455 | 0.0250 | 0.0026 | 0.0601 | 0.0530 | 0.0047 | 0.1273 | 0.0309 | 0.0003 | 0.0916 |
| inbred (overall) | 0.0318 | 0.0000 | 0.0816 | 0.0183 | 0.0000 | 0.0587 | 0.0215 | 0.0000 | 0.0849 | 0.0062 | 0.0000 | 0.0467 |
| inbred | 0.0087 | -0.0024 | 0.0328 | 0.0064 | -0.0021 | 0.0298 | 0.0052 | -0.0055 | 0.0381 | 0.0054 | -0.0059 | 0.0425 |
| symmetric epistatic | 0.0639 | 0.0150 | 0.1276 | 0.0450 | 0.0050 | 0.1054 | 0.0286 | -0.0025 | 0.1047 | 0.0333 | -0.0024 | 0.1353 |
| asymmetric epistatic | 0.0260 | 0.0045 | 0.0596 | 0.0227 | 0.0014 | 0.0592 | 0.0272 | -0.0014 | 0.0922 | 0.0268 | -0.0012 | 0.1067 |
| female (overall) | 0.0288 | -0.0003 | 0.0655 | 0.0280 | -0.0001 | 0.0661 | 0.0395 | -0.0005 | 0.1001 | 0.0393 | -0.0008 | 0.1040 |
| sex-by-additive | 0.0308 | 0.0034 | 0.0696 | 0.0168 | 0.0009 | 0.0467 | 0.0167 | -0.0010 | 0.0628 | 0.0152 | -0.0010 | 0.0679 |
| sex-by-maternal | 0.0138 | 0.0000 | 0.0373 | 0.0219 | 0.0002 | 0.0585 | 0.0286 | -0.0003 | 0.0931 | 0.0089 | -0.0012 | 0.0385 |
| female inbred (overall) | 0.0023 | -0.0026 | 0.0190 | 0.0026 | -0.0028 | 0.0210 | 0.0056 | -0.0042 | 0.0366 | 0.0040 | -0.0043 | 0.0342 |
| sex-by-inbred | 0.0053 | -0.0018 | 0.0269 | 0.0023 | -0.0014 | 0.0154 | 0.0041 | -0.0021 | 0.0462 | 0.0017 | -0.0020 | 0.0241 |
| sex-by-symm. epis. | 0.0147 | -0.0001 | 0.0447 | 0.0100 | -0.0006 | 0.0354 | 0.0132 | -0.0014 | 0.0811 | 0.0088 | -0.0014 | 0.0666 |
| sex-by-asymm. epis. | 0.0257 | 0.0017 | 0.0713 | 0.0213 | -0.0006 | 0.0734 | 0.0165 | -0.0021 | 0.1099 | 0.0091 | -0.0017 | 0.0669 |
| total explained | 0.3389 | 0.2373 | 0.4435 | 0.2866 | 0.1907 | 0.3939 | 0.4186 | 0.2488 | 0.6053 | 0.3381 | 0.1740 | 0.5365 |
| noise | 0.6611 | 0.5565 | 0.7627 | 0.7134 | 0.6061 | 0.8093 | 0.5814 | 0.3947 | 0.7512 | 0.6619 | 0.4635 | 0.8260 |

[a] To indicate the relative contribution of different diallel effects on host response to IAV, TreVarPs are given for overall effects (female, inbred, female inbred) and strain/strainpair-specific effects (additive, maternal, inbred, symmetric epistatic, asymmetric epistatic) on D1delta through D4delta (median and 95% HPD intervals).

**Table S3 Model inclusion probabilities for diallel effects classes, (A) before and (B) after accounting for *Mx1* diplotype.** We consider the following levels for strength of evidence for inclusion: positive {(0.05, 0.25] or [0.75, 0.95)}, strong {(0.01, 0.05] or [0.95, 0.99)} and very strong {[0, 0.01] or [0.99, 1]}, as described in the Statistical Methods. Part B of **Figures S7 through S14** show bar plots of posterior MIP values presented here.

| (A) Effect Class | D1 | D2 | D3 | D4 |
|---|---|---|---|---|
| theta | 1.0000 | 1.0000 | 1.0000 | 1.0000 |
| fixed: D0 | 0.0179 | 0.4630 | 0.6074 | 0.6699 |
| random: batch | 0.0772 | 0.8523 | 1.0000 | 0.9999 |
| additive | 0.0141 | 0.0494 | 0.9779 | 0.9980 |
| maternal | 0.0082 | 0.0713 | 0.7446 | 0.7400 |
| inbred (overall) | 0.3995 | 0.0164 | 0.9584 | 0.9992 |
| inbred | 0.1504 | 0.2024 | 0.3050 | 0.3504 |
| symmetric epistatic | 0.2309 | 0.3305 | 0.6674 | 0.9598 |
| asymmetric epistatic | 0.0366 | 0.1193 | 0.8197 | 0.9662 |
| female (overall) | 0.0904 | 0.1718 | 0.1719 | 0.3135 |
| sex-by-additive | 0.3140 | 0.1993 | 0.3174 | 0.6220 |
| sex-by-maternal | 0.0738 | 0.3212 | 0.5550 | 0.3697 |
| female inbred (overall) | 0.0237 | 0.0428 | 0.1012 | 0.0943 |
| sex-by-inbred | 0.2527 | 0.2325 | 0.4448 | 0.3981 |
| sex-by-symmetric epistatic | 0.1399 | 0.1694 | 0.3228 | 0.5873 |
| sex-by-asymmetric epistatic | 0.2016 | 0.3683 | 0.3682 | 0.4037 |

| (B) Effect Class | D1 | D2 | D3 | D4 |
|---|---|---|---|---|
| theta | 1.0000 | 1.0000 | 1.0000 | 1.0000 |
| fixed: D0 | 0.0180 | 0.4590 | 0.6319 | 0.5172 |
| random: batch | 0.0708 | 0.8332 | 0.9969 | 0.9979 |
| random: *Mx1* diplo | 0.0345 | 0.5515 | 1.0000 | 1.0000 |
| additive | 0.0120 | 0.0558 | 0.3835 | 0.4305 |
| maternal | 0.0068 | 0.0671 | 0.6138 | 0.5272 |
| inbred (overall) | 0.3977 | 0.0238 | 0.6696 | 0.8789 |
| inbred | 0.1420 | 0.2068 | 0.2737 | 0.3334 |
| symmetric epistatic | 0.2195 | 0.3642 | 0.5561 | 0.7668 |
| asymmetric epistatic | 0.0318 | 0.1176 | 0.6825 | 0.8511 |
| female (overall) | 0.0876 | 0.1762 | 0.2294 | 0.3394 |
| sex-by-additive | 0.3051 | 0.1981 | 0.2859 | 0.4884 |
| sex-by-maternal | 0.0662 | 0.3194 | 0.4769 | 0.2939 |
| female inbred (overall) | 0.0237 | 0.0432 | 0.0913 | 0.0889 |
| sex-by-inbred | 0.2466 | 0.2345 | 0.3658 | 0.3345 |
| sex-by-symmetric epistatic | 0.1317 | 0.1692 | 0.3028 | 0.4359 |
| sex-by-asymmetric epistatic | 0.1924 | 0.3719 | 0.3387 | 0.3296 |

**Table S4** Details of populations analyzed for additive strain-specific effects in **Figure 7**.

| POPULATION | diallel | pre-CC | CC-RIX |
|---|---|---|---|
| Recombinant | no | yes | yes |
| Inbreds | yes (some) | yes | no |
| Heterozygotes | yes (most) | no | yes |
| Sexes | males & females | females | females |
| IAV used | H1N1 (PR8) | H1N1 (PR8) | H1N1 (CA04) |
| Post-infection day analyzed | day 4 | day 4 | day 7 |
| Model | Bayes Diallel | Diploffect | Diploffect |

**Table S5** Table of CC-RIX lines used in this experiment. A total of 1,402 mice from 105 CC-RIX lines (about 13 mice per line), originating from a sparse diallel cross of 65 CC strains, were assayed for weight loss on day 7 post-infection.

| CC-RIX ID | count | CC-RIX ID | count | CC-RIX ID | count |
|---|---|---|---|---|---|
| CC001xCC055 | 16 | CC020xCC008 | 17 | CC042xCC019 | 13 |
| CC001xCC074 | 11 | CC021xCC023 | 15 | CC042xCC025 | 5 |
| CC002xCC011 | 14 | CC021xCC032 | 3 | CC043xCC033 | 21 |
| CC002xCC021 | 15 | CC022xCC024 | 11 | CC043xCC037 | 27 |
| CC003xCC051 | 12 | CC022xCC044 | 3 | CC044xCC060 | 18 |
| CC003xCC062 | 18 | CC023xCC025 | 18 | CC045xCC044 | 11 |
| CC004xCC011 | 15 | CC024xCC023 | 15 | CC046xCC068 | 21 |
| CC004xCC012 | 12 | CC024xCC052 | 11 | CC049xCC036 | 8 |
| CC005xCC001 | 15 | CC025xCC028 | 20 | CC051xCC005 | 14 |
| CC005xCC040 | 15 | CC026xCC034 | 17 | CC051xCC009 | 14 |
| CC006xCC007 | 20 | CC026xCC042 | 7 | CC052xCC007 | 3 |
| CC006xCC039 | 3 | CC027xCC036 | 3 | CC052xCC014 | 15 |
| CC007xCC070 | 12 | CC027xCC045 | 14 | CC055xCC028 | 21 |
| CC008xCC009 | 3 | CC028xCC024 | 21 | CC056xCC033 | 20 |
| CC008xCC010 | 16 | CC028xCC030 | 18 | CC057xCC052 | 16 |
| CC008xCC018 | 10 | CC029xCC027 | 16 | CC058xCC022 | 17 |
| CC009xCC040 | 12 | CC029xCC071 | 6 | CC059xCC065 | 4 |
| CC010xCC015 | 21 | CC030xCC023 | 23 | CC060xCC006 | 15 |
| CC010xCC060 | 9 | CC030xCC061 | 17 | CC061xCC025 | 16 |
| CC011xCC032 | 13 | CC032xCC013 | 18 | CC061xCC026 | 16 |
| CC011xCC042 | 15 | CC032xCC017 | 15 | CC061xCC039 | 15 |
| CC012xCC032 | 12 | CC033xCC046 | 18 | CC062xCC046 | 3 |
| CC012xCC038 | 20 | CC033xCC068 | 21 | CC062xCC072 | 3 |
| CC013xCC041 | 28 | CC034xCC016 | 10 | CC063xCC001 | 3 |
| CC014xCC059 | 4 | CC035xCC071 | 14 | CC063xCC002 | 7 |
| CC015xCC018 | 12 | CC036xCC051 | 16 | CC065xCC010 | 10 |
| CC015xCC059 | 15 | CC037xCC046 | 12 | CC065xCC072 | 1 |
| CC016xCC038 | 18 | CC037xCC057 | 19 | CC068xCC043 | 9 |
| CC016xCC061 | 13 | CC038xCC013 | 14 | CC070xCC003 | 15 |
| CC017xCC004 | 13 | CC039xCC020 | 14 | CC071xCC053 | 12 |
| CC017xCC041 | 16 | CC040xCC003 | 16 | CC072xCC063 | 4 |
| CC018xCC009 | 19 | CC040xCC015 | 13 | CC072xCC075 | 9 |
| CC018xCC065 | 16 | CC041xCC012 | 12 | CC074xCC058 | 7 |
| CC019xCC002 | 19 | CC041xCC016 | 18 | CC075xCC021 | 3 |
| CC019xCC004 | 16 | CC042xCC017 | 14 | CC075xCC035 | 14 |

**Table S6** Summary of overall and additive effects from **Figures 4A, S7A**, **S8A**, **S9A**, and **S10A**. Posterior median and 95% HPD intervals are provided for D1 through D4 p.i.

| **Diallel Effect** | **D1** | 0.025 | 0.975 | **D2** | 0.025 | 0.975 | **D3** | 0.025 | 0.975 | **D4** | 0.025 | 0.975 |
|---|---|---|---|---|---|---|---|---|---|---|---|---|
| treatment (overall) ($\theta$) | -0.131 | -0.484 | 0.223 | -0.833 | -1.328 | -0.318 | -5.594 | -6.470 | -4.729 | -8.849 | -9.920 | -7.779 |
| inbred (overall) | 1.258 | 0.407 | 2.100 | 1.212 | -0.002 | 2.413 | 0.416 | -1.641 | 2.477 | -0.524 | -3.122 | 2.018 |
| female (overall) | -0.895 | -1.447 | -0.362 | -1.245 | -2.037 | -0.460 | -1.655 | -3.066 | -0.220 | -2.108 | -3.868 | -0.295 |
| female inbred (overall) | 1.221 | -0.269 | 2.680 | 1.771 | -0.375 | 3.940 | -1.286 | -5.052 | 2.438 | -1.077 | -5.872 | 3.559 |
| fixed: D0 weight | -0.135 | -0.285 | 0.017 | -0.306 | -0.522 | -0.093 | -0.450 | -0.928 | 0.025 | -0.473 | -1.061 | 0.123 |
| additive: AJ | -0.089 | -0.641 | 0.445 | -0.016 | -0.689 | 0.647 | -2.167 | -3.721 | -0.611 | -2.767 | -4.661 | -0.864 |
| additive: B6 | -0.086 | -0.642 | 0.450 | -0.153 | -0.845 | 0.524 | -0.606 | -2.010 | 0.801 | -0.730 | -2.638 | 1.181 |
| additive: 129 | 0.270 | -0.283 | 0.840 | 0.316 | -0.379 | 1.031 | 0.002 | -1.442 | 1.462 | -0.997 | -2.878 | 0.917 |
| additive: NOD | 0.115 | -0.440 | 0.667 | 0.062 | -0.608 | 0.771 | -0.827 | -2.249 | 0.601 | -1.764 | -3.703 | 0.116 |
| additive: NZO | 0.191 | -0.363 | 0.747 | 0.276 | -0.437 | 1.021 | 2.536 | 0.719 | 4.269 | 4.066 | 1.948 | 6.121 |
| additive: CAST | -0.184 | -0.752 | 0.362 | -0.120 | -0.812 | 0.579 | 0.731 | -0.674 | 2.157 | 1.235 | -0.649 | 3.092 |
| additive: PWK | -0.149 | -0.699 | 0.411 | -0.258 | -0.967 | 0.425 | 1.698 | 0.116 | 3.228 | 4.061 | 1.971 | 6.076 |
| additive: WSB | -0.067 | -0.610 | 0.471 | -0.108 | -0.809 | 0.567 | -1.350 | -2.889 | 0.157 | -3.088 | -5.008 | -1.176 |
| $\sigma^2$ | 3.808 | 2.923 | 4.786 | 8.188 | 6.284 | 10.182 | 12.199 | 6.881 | 17.899 | 19.679 | 12.024 | 28.777 |

■ **Table S7** Summary of overall, additive, and *Mx1* effects from **Figures S11A**, **S12A**, **S13A**, and **S14A**. Posterior median and 95% HPD intervals are provided for D1 through D4 p.i.

| **Diallel Effect** | **D1** | 0.025 | 0.975 | **D2** | 0.025 | 0.975 | **D3** | 0.025 | 0.975 | **D4** | 0.025 | 0.975 |
|---|---|---|---|---|---|---|---|---|---|---|---|---|
| treatment (overall) ($\theta$) | -0.193 | -1.128 | 0.707 | -0.925 | -1.991 | 0.113 | -5.464 | -7.675 | -3.102 | -7.986 | -11.202 | -4.147 |
| inbred (overall) | 1.296 | 0.313 | 2.276 | 1.385 | 0.017 | 2.738 | 1.945 | -0.417 | 4.282 | 1.046 | -1.971 | 4.101 |
| female (overall) | -0.894 | -1.435 | -0.356 | -1.251 | -2.039 | -0.468 | -1.681 | -3.061 | -0.333 | -2.105 | -3.905 | -0.339 |
| female inbred (overall) | 1.224 | -0.231 | 2.705 | 1.765 | -0.364 | 3.931 | -1.221 | -4.945 | 2.312 | -1.018 | -5.663 | 3.654 |
| fixed: D0 weight | -0.137 | -0.289 | 0.012 | -0.313 | -0.528 | -0.099 | -0.548 | -0.996 | -0.096 | -0.576 | -1.155 | -0.016 |
| additive: AJ | -0.094 | -0.679 | 0.489 | -0.015 | -0.718 | 0.691 | -1.147 | -2.589 | 0.112 | -0.854 | -2.819 | 0.549 |
| additive: B6 | -0.086 | -0.664 | 0.501 | -0.145 | -0.867 | 0.567 | 0.190 | -0.980 | 1.352 | 0.401 | -1.017 | 1.891 |
| additive: 129 | 0.272 | -0.327 | 0.873 | 0.323 | -0.404 | 1.076 | 0.457 | -0.695 | 1.663 | 0.174 | -1.299 | 1.600 |
| additive: NOD | 0.113 | -0.453 | 0.722 | 0.067 | -0.639 | 0.799 | -0.046 | -1.221 | 1.130 | -0.148 | -1.769 | 1.266 |
| additive: NZO | 0.167 | -0.510 | 0.859 | 0.232 | -0.576 | 1.068 | 0.635 | -0.737 | 2.279 | 0.447 | -1.221 | 2.979 |
| additive: CAST | -0.137 | -0.935 | 0.619 | -0.048 | -0.942 | 0.831 | 0.267 | -1.225 | 1.866 | 0.230 | -1.545 | 2.277 |
| additive: PWK | -0.162 | -0.857 | 0.506 | -0.303 | -1.146 | 0.493 | 0.105 | -1.273 | 1.578 | 0.523 | -1.124 | 3.116 |
| additive: WSB | -0.072 | -0.653 | 0.501 | -0.111 | -0.836 | 0.605 | -0.514 | -1.811 | 0.650 | -0.909 | -2.962 | 0.533 |
| *Mx1*: *cast* × *cast* | -0.043 | -1.555 | 1.470 | -0.016 | -1.712 | 1.652 | 0.511 | -2.990 | 4.202 | 1.257 | -3.773 | 6.904 |
| *Mx1*: *cast* × *dom* | 0.057 | -1.077 | 1.193 | -0.089 | -1.397 | 1.183 | -0.311 | -3.008 | 2.256 | -1.287 | -5.596 | 2.401 |
| *Mx1*: *cast* × *mus* | -0.238 | -1.572 | 1.028 | 0.036 | -1.409 | 1.521 | 0.317 | -2.619 | 3.433 | 0.451 | -3.933 | 5.062 |
| *Mx1*: *dom* × *dom* | 0.028 | -1.076 | 1.217 | 0.015 | -1.253 | 1.290 | -2.831 | -5.872 | -0.131 | -5.274 | -9.857 | -0.779 |
| *Mx1*: *dom* × *mus* | 0.135 | -0.897 | 1.213 | 0.290 | -0.870 | 1.522 | 2.198 | -0.207 | 4.743 | 2.624 | -1.118 | 6.299 |
| *Mx1*: *mus* × *mus* | 0.061 | -1.251 | 1.445 | -0.234 | -1.817 | 1.213 | 0.016 | -3.171 | 3.295 | 1.948 | -2.574 | 7.167 |
| $\sigma^2$ | 3.797 | 2.913 | 4.769 | 8.190 | 6.328 | 10.268 | 11.270 | 6.644 | 16.627 | 19.296 | 12.210 | 27.397 |

**Table S8** Summary of dominance index for *Mx1* from *cast* or *mus*, combined with *Mx1* from *dom* in our diallel study. Posterior mode and and 80% HPD intervals are provided from D1 through D4 p.i. The posterior mode is obtained from the value of $\mathcal{D}$ that has the maximum posterior density within the range $[-1, 2]$, via the `density` function from the `stats` package in R.

| index | D1 | D2 | D3 | D4 |
|---|---|---|---|---|
| $\mathcal{D}^{(cast;\ dom)}$ | 0.726 (-1.322, 2.676) | 0.708 (-1.476, 2.833) | 0.421 (-0.534, 0.907) | 0.491 (-0.028, 0.836) |
| $\mathcal{D}^{(mus;\ dom)}$ | 0.650 (-1.181, 2.460) | 0.832 (-1.410, 3.101) | -0.278 (-2.547, 0.329) | 0.068 (-0.568, 0.381) |
| $\mathcal{D}^{(cast;\ mus)}$ | 0.468 (-2.060, 3.042) | 0.521 (-2.037, 2.966) | 0.450 (-1.884, 2.794) | 0.526 (-2.197, 3.135) |

**Algorithm 1:** Multiple Imputation Matched Quartets (MIMQ)

**Data:** Treatment diallel data frame ($T$)
**Result:** Posterior effect estimates for phenotypes in $P$

1 $P \leftarrow$ phenotypes (*e.g.* $\text{pct}^{\text{D1}}, \ldots, \text{pct}^{\text{D4}}$);
2 $D \leftarrow$ diallel categories ($\{jk, s, h\}$);
3 $R \leftarrow$ total number of replicates ($n = 1000$);
4 $C \leftarrow$ covariates;
5 **for** $p \in P$ **do**
6     **for** $d \in D$ **do**
7         Identify number of individuals to be deleted from this class (0-1); if $> 0$, add class to set of classes to have deletions ($D_{\text{delete}}$)
8         Identify number of individuals to be imputed from this class (0-3); if $> 0$ add class to set of classes to have imputation ($D_{\text{impute}}$)
9     **end**
10     **for** $r \leftarrow 1$ **to** $R$ **do**
11         Make copy of $T$: $T^{(r)}$
12         **for** $d \in D_{delete}$ **do**
13             Randomly delete row from class $d$ in $T^{(r)}$;
14         **end**
15         **for** $c \in C$ **do**
16             Obtain diallel effect ($\hat{\boldsymbol{\beta}}_c$) and variance ($\hat{\sigma}_c^2$) estimates by fitting covariate data for $c$ with BayesDiallel;
17             **for** $d \in D_{impute}$ **do**
18                 Insert row(s) for class $d$ into $T^{(r)}$, imputing $c_d$ according to the following: $\tilde{c}_d^r \sim N(\mathbf{d}\hat{\boldsymbol{\beta}}_c, \hat{\sigma}_c^2)$; based on counts from (8), multiple rows with different $c_d$ values may be inserted;
19             **end**
20         **end**
21         Obtain diallel effect ($\hat{\boldsymbol{\beta}}_p$) and variance ($\hat{\sigma}_p^2$) estimates by fitting phenotype data for $p$ with BayesDiallel, conditional on imputed covariate(s);
22         **for** $d \in D_{impute}$ **do**
23             Impute $p_d$ into phenotype column of respective $T^{(r)}$ row(s) according to the following: $\tilde{p}_d^r | \tilde{c}_d^r \sim N(\mathbf{d}\hat{\boldsymbol{\beta}}_p, \hat{\sigma}_p^2 | \tilde{c}_d^r)$
24         **end**
25         Run BayesDiallel on $T^{(r)}$;
26     **end**
27     Concatenate MCMC results from each of the $T^{(r)}$ runs and summarize;
28 **end**
